# Isolation of bioactive compounds from *Bergenia ciliata* (haw.) Sternb rhizome and their antioxidant and anticholinesterase activities

**DOI:** 10.1186/s12906-019-2679-1

**Published:** 2019-11-06

**Authors:** Roheena Zafar, Habib Ullah, Muhammad Zahoor, Abdul Sadiq

**Affiliations:** 1 Department of Chemistry, University of Malakand, Chakdara, Dir Lower, KPK 18800 Pakistan; 2 Department of Pharmacy, Sarhad University of Science and Information Technology, Peshawar, Pakistan; 3 Department of Pharmacy, University of Malakand, Chakdara, Dir Lower, KPK 18800 Pakistan

**Keywords:** *Bergenia ciliata* rhizome, Total phenolic contents, Total flavonoid contents, Antioxidants, Anticholinesterase assay, Pyrogallol, Rutin, Morin

## Abstract

**Background:**

*Bergenia ciliata* is a medicinal plant used for the treatment of diarrhea, vomiting, fever, cough, diabetes, cancer, pulmonary disorders and wound healing.

**Methods:**

In this study, *Bergenia ciliata* crude extract, subfractions, and isolated compounds were evaluated for their antioxidant and anticholinesterase potential. The free radical scavenging capacities of the extracts determined using DPPH and ABTS assays. The anticholinesterase potentials were determined using acetylcholine esterase and butyryl choline esterase enzymes. To determine the phytochemical composition, the extracts were subjected to HPLC analysis and silica gel column isolation. Based on HPLC fingerprinting results, the ethyl acetate fraction was found to have more bioactive compounds and was therefore subjected to silica gel column isolation. As a result, three compounds; pyrogallol, rutin, and morin were isolated in the pure state. The structures of the isolated compounds were elucidated using spectroscopic techniques like ^1^H-NMR, IR and UV-Visible.

**Results:**

The crude extract showed maximum anticholinesterase (acetylcholinesterase = 90.22 ± 1.15% and butyrylcholinesterase = 88.22 ± 0.71%) and free radical scavenging (87.37 ± 2.45 and 83.50 ± 0.70% respectively against DPPH and ABTS radicals) potentials. The total phenolic contents (expressed as equivalent of gallic acid; mgGAE/g) were higher in ethyl acetate fraction (80.96 ± 1.74) followed by crude extract (70.65 ± 0.86) while the flavonoid contents (expressed as quercetin equivalent; mgQE/g) and were higher in crude extract (88.40 ± 1.12) followed by n-butanol fraction (60.10 ± 1.86). The isolated bioactive compounds pyrogallol, rutin, and morin were found active against ABTS and DPPH free radicals. Amongst them, pyrogallol was more active against both free radicals. Reasonable anticholinesterase activities were recorded for pyrogallol against selected enzymes.

**Conclusion:**

The extracts and isolated compounds showed antioxidant and acetylcholinesterase inhibitory potentials. It was concluded that this plant could be helpful in the treatment of oxidative stress and neurological disorders if used in the form of extracts.

## Background

For thousands of years, plants have been used as sources of medicines by mankind. According to the World Health Organization, even today about 80% of the world population depends on traditional medication from plants as they are factories of natural phytochemicals [1]. Phenolic compounds are an important group amongst the phytochemicals naturally prepared by plants [2]. Most of the phenolic compounds have antioxidant properties and help in the prevention of heart diseases, reduction of inflammation, lowering the incidence of diabetes, cancers and mutagenesis in human [3]. The interest in the isolation of antioxidant from medicinal plants has been increased many folds from the last two decades as they have minimum incidences of side effects and are widely used in scientific research and industries [4]. A number of free radicals are constantly produced inside the human body during normal metabolic processes. About 1/4th of the oxygen inhaled is converted into free radicals. They are very reactive and causes a number of health complications. Antioxidants have the potential to scavenge them and maintain health [5–7].

A number of synthetic antioxidants like butyl hydroxyanisole and butyl hydroxytoluene have been used in food products as preservatives. However, the toxicological studies on butyl hydroxyanisole and butyl hydroxytoluene have shown that they can induce impairment of blood clotting in the experimental animal. The butyl hydroxytoluene has toxic effects on the lungs as well. Butylhydroxyanisole can induce tumor in the forestomach of experimental animals whereas long term exposure to butylhydroxytoluene can induce liver tumor in them [8]. Due to the side effects of synthetic antioxidants, the use naturally occurring antioxidants in pharmaceutical products, foods, and cosmetics industries have been increased [9–11]. Studies in this regards are needed to determine the antioxidant power of naturally occurring compounds and to develop efficient methods for their extraction, and isolation.

A number of useful secondary metabolites like gallic acid, tannic acid, glucose, mucilage, bergenin, stigmasterol, β-sitosterol, arbutin, phytol, damascenone, 3-methyl-2-buten-1-ol etc. have been isolated from *Bergenia ciliata, Bergenia ligulatas* and *Bergenia stracheyi* [12–14]. Various parts of these species are used to remove urinary bladder stone and have shown antilithics, diuretic, anti-bradykinin, antiviral, antipyretic, antibacterial, anti-inflammatory, hepatoprotective, insecticidal and α-glycosidase activities [11]. Amongst the mentioned three species of genus Bergenia, *Bergenia ciliata* is widely used in folk medicines and have exhibited antitussive, antiulcer, antioxidant, antibacterial, hypoglycemic, toxicological anticancer and ant-adiabatic activities [12].

The high demand and importance of natural antioxidant from plants origin promoted us to evaluate the *Bergenia ciliata* rhizome anticholinesterase and antioxidant potentials. First the crude extract and sub fractions were screened for their antioxidant and cholinesterase inhibitory potentials. The extracts were then subjected to HPLC analysis for the identification of phenolic compounds present in them. Based on HPLC finger printing results ethyl acetate fraction was subjected to silica gel column isolation that led to isolation of three bioactive compounds: pyrogallol, rutin and morin. The isolated compounds were characterized by FTIR and NMR.

## Methods

### Chemicals, drugs and standards

The antioxidant standards: quercetin, morin, rutin, pyrogallol, mandalic acid, hydroxy benzoic acid, phloroglucinol and chlorogenic acid were obtained from Sigma-Aldrich France. From the same firm galanthamine (from Lycoris Sp.) was also obtained. Follin-ciocalteu reagent, sodium carbonate, 5,5-dithio-bis-nitrobenzoic acid (DTNB) and 2,2-Diphenyle-1 picrylhydrazyle (DPPH) were purchased from Sigma Aldrich CHEMIE GmbH USA. Aluminum chloride, sodium nitrite, sodium hydroxide, ethanol, methanol, ascorbic acid, 2,2′-azino-bis-3 ethylbenzothiazoline-6-sulfonic acid (ABTS) were bought from sigma Aldrich Germany. Phosphate buffer (pH 8), butyryl thiocholine iodide, acetylthiocholine iodide, acetyl cholinesterase from Electric eel (type-VI-S) and butyryl cholinesterase from equine used in enzyme inhibition assays were obtained from Sigma-Aldrich USA. All the chemicals used were of analytical grade except the HPLC solvents which were HPLC grade and were purchased from DaeJung Korea. They were used as such without any further purification. Distilled water used for HPLC analysis and antioxidant assays were prepared in biochemistry lab using automatic water still (Daihen labtech, china). The HPLC solvents were subjected to sonication (Elmasonic, Model E 30 H) to remove air bubbles before use.

### Plant sample collection

**Fig. 1:**
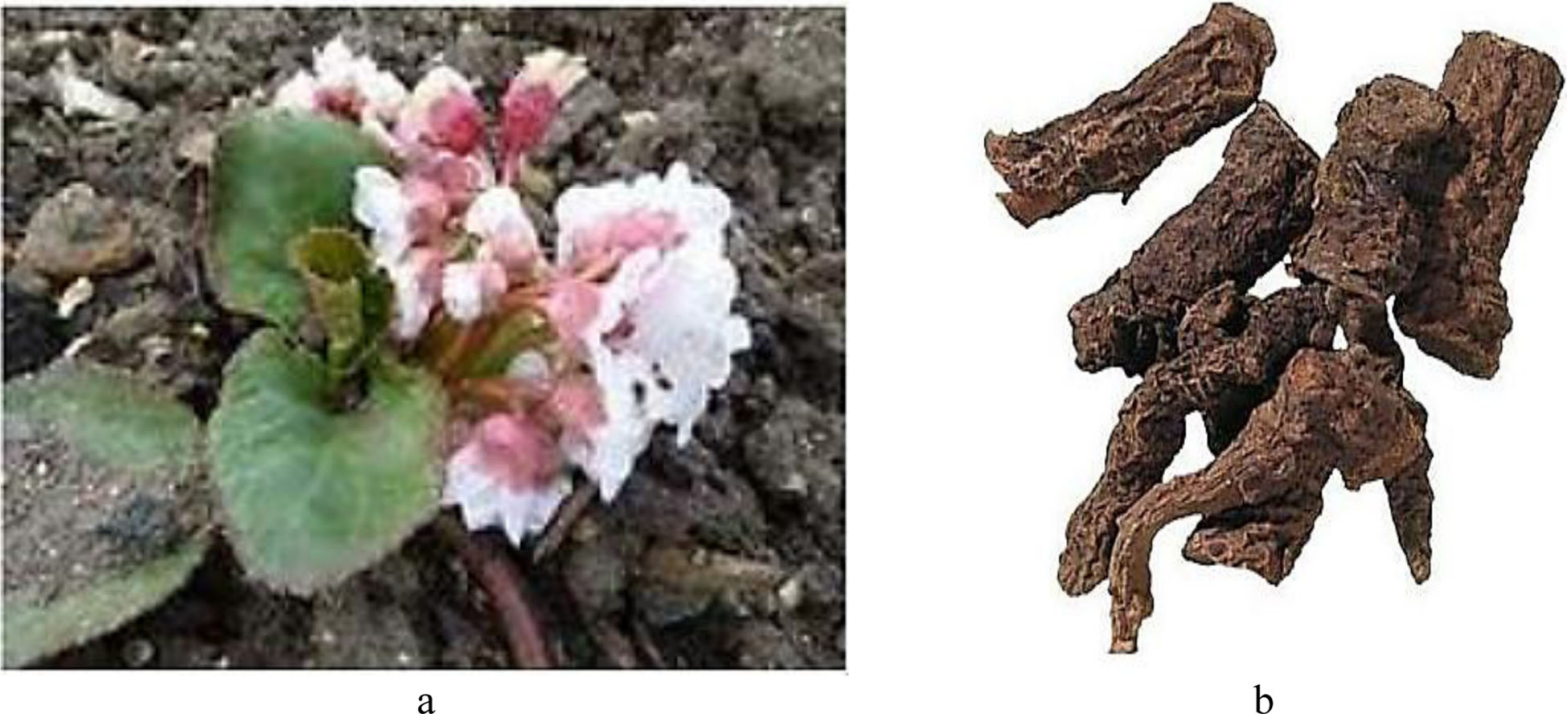
Bergenia ciliata a = whole plant, b = part of the plant used in this study (rhizome)

The rhizomes of the *Bergenia ciliata* (Fig. 1) were collected from Laram Mountain, Dir Lower, KPK province of Pakistan in January 2015. The plant was authenticated by Dr. Nisar Department of Botany University of Malakand. A voucher specimen (1019HU) was also deposited in the Herbarium of Malakand University.

### Extraction and fractionation

About 5 kg of fresh rhizomes of the *B. ciliata* were thoroughly washed with tap water to remove dust and soil particles and were kept in a shady place at room temperature for 4 weeks. The dried sample mass obtained after drying was 2.05 kg which was then grinded into fine powder. To obtain crude extract, the powder sample was soaked in 90% methanol (8 L) for 72 h. The mixture was then filtered using Whattman filter paper. The residue left was dipped again in methanol for 72 h and the filtrates from this step were then combined with the previous one. The filtrates were evaporated in rotary evaporator (Rota vapor R-200 Buchi, Switzerland) at 45 °C under reduced pressure. The semisolid mass obtained was then kept in open atmosphere to evaporate the remaining solvent. The final mass of crude solid extract was 380 g. Fractionation of the crude extract was done according to the described protocol of Haq et al. [12]. Appropriate amount (350 g) of crude extract was taken and dissolved it in 900 ml of distilled water and subjected to solvent-solvent extraction. All extracts were analyzed through reverse phase HPLC for the identification of possible phenolic antioxidants. The ethyl acetate fraction was rich in bioactive compounds and was subjected to silica gel column isolation.

### Determination of total phenolic content

The total phenolic content in *Bergenia ciliata* rhizome crude extract and sub fractions were determined using Follin-ciocalteu assay with a little modification [14]. For the preparation of extracts standard solutions, 5 mg of each extract were dissolved in 5 ml methanol. About 1 ml of Follin-ciocalteu reagent was taken and diluted to 10 ml with distilled water. Working standards of each extract were prepared by mixing 1 ml (each) standard solution with 9 ml distilled water. To each test tube, 1 ml of diluted Follin-ciocalteu reagent was added and allowed to stand for 6 min. To reaction mixtures in each test tube then 10 ml of 7% sodium carbonate solution was added and diluted further to 25 ml by the addition of distilled water and incubated for 90 min at room temperature. The absorbance of the samples were recorded at 760 nm using UV spectrophotometer. For the determination of total phenolic contents a calibration curve of gallic acid (0 to 100 mg/ml) was drawn. The total phenolic contents were expressed as milligrams of gallic acid equivalent (mg GAE/g) per gram of dry sample.

### Determination of total flavonoid content

For the determination of total flavonoid contents in *Bergenia ciliata* rhizome extracts, the procedure described by Park et al. was followed [15] with little modification. About 5 mg of each of the extracts were dissolved in 5 ml of methanol. From each of these standard solutions, 1 ml were mixed with 9 ml distilled water and finally with 1 ml NaNO_2_ (5%). The mixtures were allowed to stand for 6 min to proceed the reaction. Then 2 ml of 10% aluminum chloride solution was added to each and allowed to stand for 5 min. Then 2 ml sodium hydroxide (1 M) was added sequentially to the mixtures. Finally, the absorbance of the mixture at 510 nm was recorded using UV spectrophotometer. For the determination of total flavonoids content standard quercetin curve (0 to 200 mg/ ml) was drawn. The total Flavonoid contents were expressed as quercetin equivalent in mg. QE/g of the dry sample.

### Determination of free radical scavenging activities using DPPH assay

The free radical scavenging abilities of the extracts were determined using 2,2- Diphenyl-1-picrylhydrazyl (DPPH) assay modified by Brand-Williams et al. with some modification [16]. In oxidized form of DPPH have deep violet color. On getting electrons from antioxidants the deep violet color changes to yellow color. DPPH solution was prepared by dissolving 20 mg in 100 ml methanol (stock solution). From this solution 3 ml were taken and its absorbance was adjusted to 0.75 at 515 nm (control solution). For the formation of free radical, the stock DPPH solution was covered with aluminum foil and kept in dark place for 24 h. For the preparation of stock solutions, 5 mg of each extracts were dissolved in 5 ml of methanol (5000 μg/5 ml). Different dilution (1000, 500, 250, 125 and 62.5 μg/ml) were prepared from stock solutions through serial dilution. About 2 ml from each dilution were mixed with 2 ml of DPPH solution and incubated for 15 min in darkness. The percentage inhibition of DPPH free radical by extracts was calculated using the following formula:

1 $$ \% inhibition=\frac{A-B}{A}X100 $$

Where A is the absorbance of DPPH in oxidized state and B is the absorbance of mixture after 15 min of reaction. Ascorbic acid was used as standard antioxidant.

### ABTS free radical scavenging assay

The 2, 2-azinobis (3-ethylbenzthiazoline)-6-sulfonic acid (ABTS) free radicals scavenging assay was used to determine the antioxidant potential of the *Bergenia ciliata* rhizome extracts, following the standard procedure devised by Re et al. [17]. ABTS (7 mM) and potassium per sulphate (2.45 mM) solutions were prepared in 100 mL methanol. These two solutions were mixed thoroughly and kept in dark overnight for the formation of free radical. About 3 ml were taken from this stock solution and its absorbance was adjusted to 0.76 at 745 nm (control solution). About 300 μl of test samples were mixed with 3 ml of ABTS solution and incubated for 15 min at 25 °C. The absorbance of the mixture was measured using a double beam spectrophotometer at 745 nm. The same procedure was followed for the preparation of different dilution of ascorbic acid (positive control). The data was recorded in triplicate and percent ABTS free radicals scavenging activities were calculated using Eq. 1.

### Anticholinesterase assays

Acetyl cholinesterase (AChE) and butyryl cholinesterase (BChE) were used to evaluate the enzyme inhibitory potential of *Bergenia ciliata* rhizome extracts and isolated compounds using Ellman’s assay [18]. The mentioned enzymes when act on their respective substrates acetylthiocholine iodide and butyrylthiocholine iodide hydrolyze them which then on complexation with 5-thio-2-nitrobenzoate anion formed from DTNB give yellow color. The color change is recorded after 15 min by spectrophotometer. Phosphate buffer (pH = 8) was prepared by mixing 1.36 g/100 mL, potassium dihydrogen phosphate and 1.74 g/100 mL di-potassium hydrogen phosphate (6 and 94% respectively). The AChE solution was prepared by dissolving 0.5 mg of enzyme in 0.5 ml of phosphate buffer to get 1000 unit/ml and BChE solution was also prepared by dissolving 1 mg in 1 ml of phosphate buffer to get 10 unit/ml. These two solutions were further diluted by phosphate buffer till a final concentration of 0.03 unit/ml and 0.1 unit/ml respectively. DTNB solution (0.0002 M) and ATChI and BTChI (0.0005 M) were prepared and kept in reagent bottles in refrigerator before use.

#### Spectroscopic analysis

For spectroscopic determination of the mentioned enzyme inhibition, 1 ml of crude extract and sub fractions were taken from dilutions (125–1000 μg/ml) in test tubes. To each test tube, 100 μl of enzyme and DTNB solutions were added and incubated for 15 min at 25 °C. After this 100 μl substrates (AChEI, BChEI) were added to each test tube and allowed to stand for 15 min. The absorbance of mixtures were recorded at 412 nm. A negative control was prepared by mixing all the above mention components except plant extracts. Galanthamine was used as a positive control. The same procedure was used for the preparation of Galanthamine (standard) solutions as well. For each sample absorption was recorded for 4 minutes. Percent activities of enzyme and percent inhibition were calculated using following relations.

2 $$ V=\frac{\Delta  Abs}{\Delta  T} $$

3 $$ \% enzyme\ activity=\frac{V}{Vmax}x100 $$

Where V is the rate of reaction in the presence of inhibitor and V_max_ is the rate of reaction without inhibitor.

### Determination of phenolic contents through HPLC

#### Preparation of sample and standards

For the identification of phenolic compound in *Bergenia ciliata* rhizome the previously reported method devised by Zeb was used [19]. About 1 g of powder plant sample was mixed with 10 ml methanol and water (1:1; 10 ml; v/v) mixture and vortexed for 15 min. The mixture was shaken for 1 h and then filtered with Whattman filter (pore size of 0.7 μm). The mixture was then centrifuged at 4000 rpm for 15 min. The supernatant were filtered again through PTFE Agilent (pore size 0.45 μm). The filtrate were collected into 2 ml HPLC vials and were labeled with proper code and place in refrigerator till further use. Standard solutions of quercetin, morin, rutin, mandalic acid, pyrogallol, hydroxy benzoic acid, phloroglucinol and chlorogenic acid having concentrations 90 ng each were prepared in methanol.

#### HPLC-UV conditions

The HPLC system used was an Agilent 1260 having basic parts like quaternary pump, auto sampler, degasser and ultra violet (UV) detector. The separation was achieved via Agilent Zorbax Eclipse C18 column (4.6*200 mm, 5 μm). The gradient system consisting of solvent A (methanol, acetic acid, deionized water, 10:2:88, v/v and solvent B (methanol, acetic acid, deionized water, 90:2:8, v/v). With 100% A, the efficient gradient program was started at 0 min, 85% A at 5 min, 50% A at 20 min, 30%A at 25 min, and 100% B from 30 to 40 min. The flow rate of sample was 1 ml/min. The identification of phenolic compounds were done by comparing the retention times of particular component in sample chromatogram with that of the available standards (chromatogram), while from % peaks area; quantification of the identified compounds were made.

### Isolation of phenolic compounds through column chromatography

The HPLC Chromatograms of sub fractions were compared for presence of bioactive compounds. Amongst them the ethyl acetate fraction was rich in phenolic compounds and was subjected to silica gel column isolation. Silica gel was used as adsorbent in the large and pencil column for the separation of active components. For packing of column the silica gel was suspended in required solvent for 3 h to swell and after this it was introduced into the column with care. Little amount of the ethyl acetate fraction was dissolved in a suitable solvent and was mixed with silica gel to form slurry. After mixing well the slurry was allowed to dry for 48 h. The dried slurry was then grinded to fine powder and loaded to column in such a way that the top of the column was not disturbed. The column was eluted with suitable solvent system comprising of the ethyl acetate and n-hexane mixed in different proportions. The solvent flow was controlled by peristaltic pump (SEKO Italy) to facilitate the elution. The elution was started from *n*-hexane followed by increase in polarity of *n*-Hexane/ethyl acetate gradients up to 50% ethyl acetate/n-hexane (1:1) gradient that afforded 20 sub-fractions (C_1_-C_20_). Based on TLC results, fractions were combined according to their separation profile. All these sub fractions were then re-chromatogram using pencil column. The isolated compounds were then subjected to HPLC, FTIR and NMR analysis for purity and structural elucidation.

### Statistical analysis

Each experiment was performed in triplicates and values were expressed as mean ± SEM. Two- way ANOVA followed by multiple comparison Bonferroni’s test was applied for the comparison of positive control with the test groups. The *P* values less than or equal to 0.05 were considered as statistically significant. IC_50_ values were calculated by linear regression analysis among the percent inhibition against the extracts and isolated compound concentrations via Excel program. Regression (y) and linear correlation (R^2^) for phenolic and flavonoids contents and various biological activities such as anticholinesterase and antioxidants were determined using Microsoft Excel 2007.

## Results and discussion

### Total phenolic and flavonoid contents

The phenolic and flavonoid contents in crude extract and sub fractions of *Bergenia ciliata* rhizome are presented in Table 1. The highest phenolic contents were recorded for ethyl acetate fraction (80.96 ± 1.74) follow by n-butanol (63.49 ± 2.04), crude (70.65 ± 0.86), chloroform (53.59 ± 0.80), aqueous (42.83 ± 1.43), and n-hexane (23.00 ± 1.21) extracts. The phenolic contents were expressed as Gallic acid equivalent (mgGAE/g of dry sample). The total flavonoid contents (Table 1) were highest in crude extract (88.40 ± 1.12) followed by sub fractions in the following order; n-butanol fraction (72.70 ± 0.87) > chloroform (60.10 ± 1.86) > aqueous (39.84 ± 1.12) > ethyl acetate (26.30 ± 1.08) > n-hexane (12.94 ± 1.18). They were expressed as quercetin equivalent (mg QE/g of dry sample).

**Table 1:** Total phenolic and Flavonoid content in *Bergenia ciliata* rhizome crude extract and their different subtractions

Samples	Total phenolic (mg GAE/g) of dry sample)	Sample	Total flavonoid (mg qe/g) of dry sample
Ethyl acetate	80.96 ± 1.74	crude	88.40 ± 1.12
Crude	70.65 ± 0.86	n-butanol	72.70 ± 0.87
n-butanol	63.49 ± 2.04	Chloroform	60.10 ± 1.86
Chloroform	53.59 ± 0.80	aqueous	39.84 ± 1.12
Aqueous	42.83 ± 1.43	Ethyl acetate	26.30 ± 1.08
n-hexane	23.00 ± 1.21	n-hexane	12.94 ± 1.18

*GAE* Gallic acid equivalent, *QE* Quercetin equivalent each value in the table is represented as mean ± SEM (*n* = 3)

### DPPH/ABTS radical scavenging activities

**Fig. 2:**
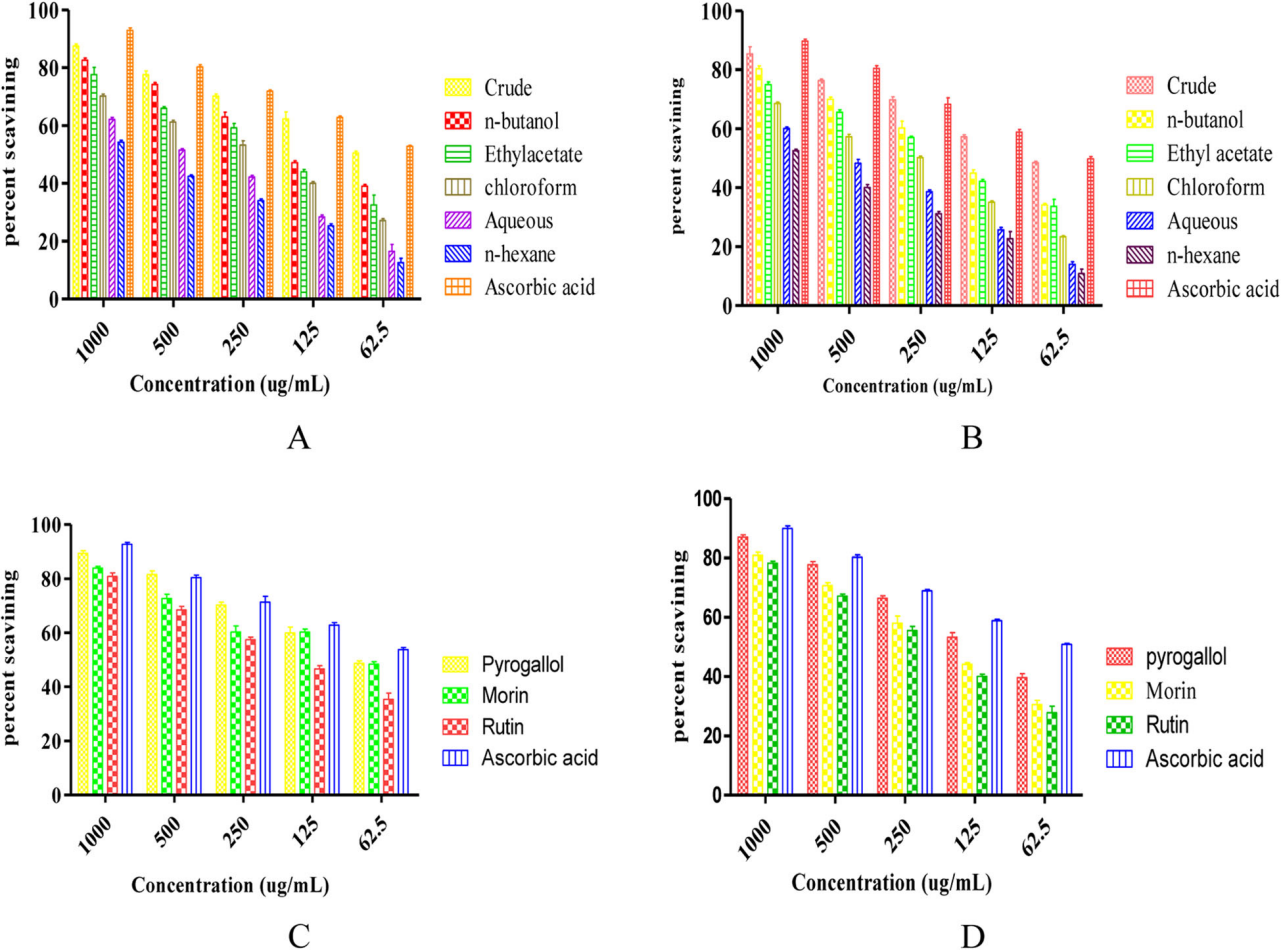
% DPPH/ABTS free radical scavenging potential of *B. ciliata* rhizome extracts and isolated compounds (A = DPPH scavenging potentials of extracts. B = ABTS scavenging potentials of extracts. C = DPPH scavenging potentials of isolated compounds, C = ABTS scavenging potentials of isolated compounds)

The antioxidant potential of Crude extract and sub-fractions of the rhizome of *Bergenia ciliata* were studied using DPPH and ABTS assays. The percent free radical scavenging potential the crude extract was higher (IC_50_ - = 2 μg/ml) than the sub fractions. Amongst the fractions n-butanol was most potent with IC_50_ value of 122 μg/ml. The IC_50_ values of other fractions; ethyl acetate, chloroform, aqueous and n-hexane were 170, 205, 440, and 830 μg/ml respectively (Table 2 and Fig. 2a). Ascorbic acid was used as a standard and its IC_50_ value was 50 μg/ml.

**Table 2:** Percent DPPH and ABTS radical scavenging potential of crude extract and their sub fractions of *Bergenia ciliata* rhizome using ascorbic acid as standard

Samples	Concentrations (μg/mL)	DPPH Percent inhibition (mean ± S.E.M)	DPPH IC_50_ (μg/mL)	ABTS percent inhibition (mean ± S.E.M)	ABTS IC_50_(μg/mL)
Crude	1000	87.50 ± 0.70*		85.37 ± 2.45 ^ns^	
	500	77.66 ± 1.20 ^ns^		76.33 ± 0.67 ^ns^	
	250	70.31 ± 0.76 ^ns^	62	69.00 ± 1.00 ^ns^	70
	125	62.34 ± 2.45 ^ns^		57.33 ± 0.66 ^ns^	
	62.5	50.50 ± 0.70 ^ns^		48.45 ± 0.52 ^ns^	
n-butanol	1000	82.62 ± 0.76***		80.26 ± 1.03***	
	500	74.32 ± 0.62**		70.05 ± 0.77***	
	250	63.00 ± 1.73***	122	60.30 ± 2.33***	140
	125	47.22 ± 0.66***		44.97 ± 1.09***	
	62.5	39.03 ± 0.60***		34.10 ± 0.50***	
Ethyl acetate	1000	77.66 ± 2.45***		75.00 ± 0.88***	
	500	66.07 ± 0.56***		65.66 ± 0.81***	
	250	59.33 ± 1.46***	170	57.02 ± 0.44***	186
	125	44.08 ± 0.73***		42.11 ± 0.66***	
	62.5	32.50 ± 3.45***		27.64 ± 2.39***	
Chloroform	1000	70.27 ± 0.71***		68.84 ± 0.47***	
	500	61.31 ± 0.60***		57.33 ± 0.78***	
	250	53.33 ± 1.45***	205	50.22 ± 0.46***	267
	125	40.05 ± 0.56***		35.01 ± 0.52***	
	62.5	27.12 ± 0.66***		23.25 ± 0.38***	
Aqueous	1000	62.18 ± 0.67***		60.15 ± 0.44***	
	500	51.50 ± 0.55***		48.33 ± 1.33***	
	250	42.02 ± 0.57***	440	37.64 ± 0.58***	600
	125	28.37 ± 0.65***		24 .62 ± 0.85***	
	62.5	16.56 ± 2.38***		13.03 ± 0.86***	
n-hexane	1000	54.27 ± 0.72***		52.50 ± 0.50***	
	500	42.49 ± 0.50***		40.00 ± 1.08***	
	250	34.07 ± 0.58***	830	31.16 ± 0.70***	910
	125	25.31 ± 0.67***		22.06 ± 2.38***	
	62.5	12.66 ± 1.45***		10.04 ± 1.54***	
Ascorbic acid	1000	92.94 ± 0.86		89.67 ± 0.73	
	500	80.29 ± 0.79	50	80.45 ± 0.96	64
	250	71.93 ± 0.45		68.34 ± 2.16	
	125	62.90 ± 0.48		58.90 ± 0.85	
	62.5	52.88 ± 0.32		49.78 ± 0.76	

Ascorbic acid was used as a positive control. Data is represented as mean ± S.E.M; (n = 3). Values significantly different as compared to positive control,*: *P* < 0.05, ***: *P* < 0.001, ns: *P* > 0.05

Against ABTS free radical again Crude extract was more potent with IC_50_ = 70 μg/ml (Table 2 and Fig. 2b). Amongst sub fractions, n-butanol fraction showed promising antioxidant activity with IC_50_ = 140 μg/ml, followed by ethyl acetate, chloroform, aqueous and n- hexane with 1C _50_ of 186, 267, 650 and 910 μg/ml respectively.

The already phytochemical screening reports on *Bergenia ciliata* revealed that it contains a number of phyto-constituent like flavonoid, steroids, terpenoids, tannin, saponins, anthraquinones and glycoside responsible for their antioxidant potentials [20, 21].

### Acetyl cholinesterase inhibition assays

The cholinesterases; AChE and BChE are the key enzymes in the breakdown of acetylcholine and butyrylcholine. In some pathological conditions (neurological disorders like taxia, dementia, Alzheimer disease) there is need to inhibit these enzymes. The decreased level of acetylcholine within the nervous system of the body may be due to reduced acetyl-transferase or increased level of AChE. The hydrolysis of acetylcholine can be minimized by inhibiting AChE in the brain. A number of plants have been tested to enhance and alleviate the cognitive function and symptoms associated with Alzheimer disease [22]. The AChE and BChE percent inhibition and their 1C_50_ values extracts are given in Table 3. The crude extract and sub fraction showed promising inhibition of acetyl cholinesterases. The highest percent inhibition of AChE was recorded for crude extract (90.22 ± 1.15, 80.15 ± 0.70, 71.02 ± 0.73, 57.26 ± 0.42 respectively for the studied concentrations: 1000, 500, 250, and 125 μg/ml) with IC_50_ = 72 μg/ml. Amongst the fractions, n-butanol fraction showed high percent inhibition (82.33 ± 1.07, 70.23 ± 3.31, 58.07 ± 1.20 and 49.03 ± 0.75 for the afore mentioned concentrations) with IC_50_ value of 140 μg/ml followed by ethyl acetate fraction. All fractions showed concentration dependent activities. The IC_50_ calculated for chloroform, aqueous and n-hexane were: 311, 400, and 560 μg/ml respectively.

**Table 3:** Percent AChE and BChE inhibition potentials of *Bergenia ciliata* Rhizome Crude extract and their sub fractions

Sample	Concentration (μg/ml)	Percent AChE (mean ± SEM)	AChE IC_50_ (μg/ml)	Percent BChE (mean ± SEM)	BChE IC_50_ (μg/ml)
Crude	1000	90.22 ± 1.15 ^ns^		88.22 ± 0.71**	
	500	80.15 ± 0.70**	72	80.47 ± 0.86 ^ns^	85
	250	71.28 ± 0.73 ^ns^		67.90 ± 3.30***	
	125	57.26 ± 0.42**		56.75 ± 2.25 ^ns^	
n-Butanol	1000	82.33 ± 1.07***		80.43 ± 1.15***	
	500	70.23 ± 3.31***	140	71.80 ± 0.90***	
	250	58.67 ± 1.20***		61.20 ± 0.65***	150
	125	49.93 ± 0.75***		48.83 ± 2.33***	
Ethyl acetate	1000	75.32 ± 2.45***		73.26 ± 0.60***	
	500	66.34 ± 0.60***		60.31 ± 0.81***	
	250	53.66 ± 1.17***	185	50.33 ± 2.33***	215
	125	44.76 ± 0.73***		35.85 ± 0.68***	
Chloroform	1000	66.69 ± 0.45***		69.19 ± 0.72***	
	500	57.39 ± 3.26***		60.66 ± 0.94***	
	250	46.19 ± 1.08***	311	45.75 ± 0.42***	350
	125	36.80 ± 0.73***		34.69 ± 0.49***	
Aqueous	1000	60.45 ± 2.33***		65.37 ± 0.50***	
	500	55.63 ± 1.40***		51.27 ± 0.81***	
	250	43.11 ± 0.78***	400	37.63 ± 0.46***	470
	125	31.66 ± 0.76***		24.31 ± 2.42***	
n-Hexane	1000	50.54 ± 1.33***		60.66 ± 0.60***	
	500	45.80 ± 2.26***	650	47.56 ± 0.60***	
	250	30.38 ± 0.47***		33.46 ± 0.88***	560
	125	18.66 ± 2.30***		20.38 ± 0.68***	
Galanthamine	1000	95.32 ± 0.88		94.50 ± 0.71	
	500	87.74 ± 0.55	45	84.66 ± 1.20	60
	250	76.44 ± 0.60		76.72 ± 0.72	
	125	64.58 ± 0.54		60.83 ± 0.69	

Galanthamine was used as a positive control. Data is represented as (mean ± S.E.M) *n* = 3. Values significantly different as compared to positive control, *: *P* < 0.05, **: *P* < 0.01, ***: *P* < 0.001, ns: *P* > 0.05

Against BChE the crude extract again showed highest potential with activities: 88.22 ± 0.71, 80.47 ± 0.86, 67.10 ± 3.30, and 56.75 ± 2.25 at studied concentrations of 1000, 500, 250 and 125 μg/ml (IC_50_ = 80 μg/ml). Amongst the sub fractions n-butanol fraction was more potent with activities of 80.00 ± 1.5, 71.80 ± 0.90, 61.12 ± 0.65, and 48.80 ± 80 at studied concentrations with IC_50_ value of 150 μg/ml. The IC_50_ values of ethyl acetate, chloroform, n-hexane and aqueous fractions were; 350, 470, and 560 μg/ml respectively (Table 3). The inhibition was dose dependent.

### Linear correlation of total phenolic and flavonoid contents vs antioxidant and anticholinesterase activities

**Fig. 3:**
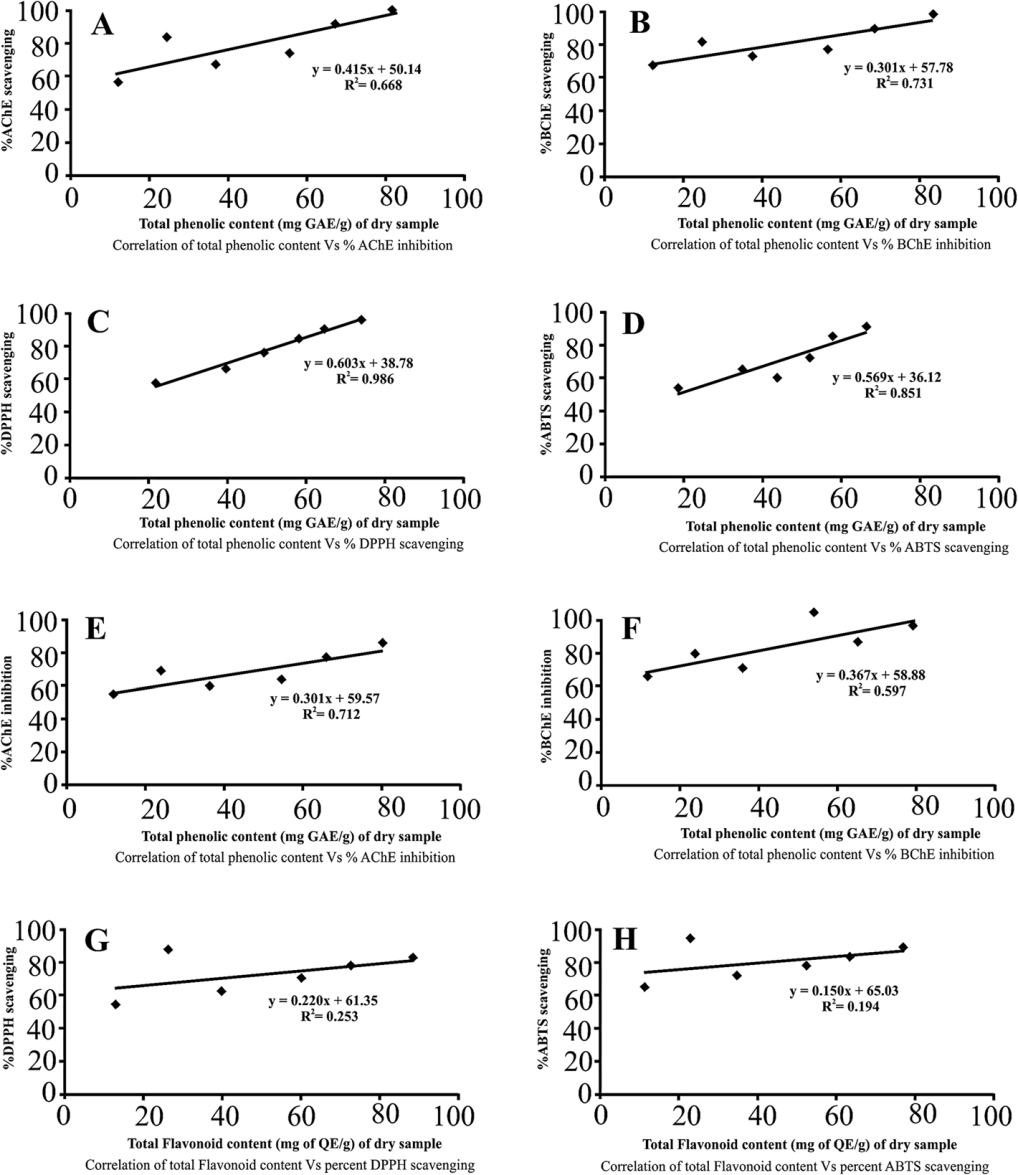
Linear correlations for total Phenolics Vs AChE (**a**), BChE (**b**), DPPH (**c**), and ABTS (**d**) and for total Flavonoid content Vs AChE (**e**), BChE (**f**), DPPH (**g**), and ABTS (**h**) activities

A linear correlation of total phenolic and flavonoid content vs various biological activities such as antioxidant (DPPH, ABTS) and anticholinesterase (AChE, BChE) have been shown in Fig. 3. The regression value of %AChE and % BChE inhibition vs total phenolic contents (Fig. 3a and b) were 0.66 and 0.73 respectively while that of % DPPH and ABTS scavenging vs TPC (Fig. 3c and d) were 0.98 and 0.85 respectively. The regression values of % AChE and BChE VS TFC (Fig. 1e and f) were 0.71 and 0.58 while that of % DPPH and ABTS vs TFC (Fig. 3g) were 0.25 and 0.194 respectively. Comparatively, good correlations of the studied biological activities have been observed for TPC while that of TFC it was poor. It was concluded that the anticholinesterase and antioxidant capacities were due to phenolic constituents.

### Phenolic composition

The phenolic compounds present in extracts were identified by comparing the HPLC chromatograms of samples with that of standards. The identification was based on comparison of retention times of the particular component with the available standards or by comparing with those reported in the literature. Those peaks were selected for quantification where the spectral purity was higher than 96%. The quantification of the identified compounds were made by single point calibration method. Quercetin, morin, rutin, pyrogallol, hydroxy benzoic acid, phloroglucinol, mandalic acid, and chlorogenic acid were used as standard phenolic compounds (Additional file 1: Figure S1).

#### Phenolic compounds in *Bergenia ciliata*

HPLC chromatogram of *Bergenia ciliata* Rhizome extracts were obtained using Agilent 1260 system. The HPLC chromatograms of crude and ethyl acetate fractions are given in Additional file 1: Figures S2 and S3 while the detail identification of compounds is given in Table 4. In crude extract, compound **2**, eluted at retention time 10.22 min, was identified as quercetin (0.46 mg/100 g) and the compound **6**, eluted at retention time 30.88 min was mandalic acid with a concentration of 0.013 mg/ 100 g. The quantification was done through the following formula:

4 $$ Cx=\frac{Ax\ast Cs\left(\raisebox{1ex}{$\mathrm{mg}$}\!\left/ \!\raisebox{-1ex}{$ ml$}\right.\right)\ast V(ml)}{As\ast Sample\ \left( wt\  in\ g\right)} $$

**Table 4:** Identification and quantification of possible phenolic compounds in *Bergenia ciliata* rhizome crude extract and ethyl acetate fraction using reversed phase HPLC

Peak	Retention time (min)	Possible identity	Quantity (mg per100g) of sample	Identification reference
2	10.22	Quercetin	0.46	Standard
3	12.76	Morin	3.13	Standard
4	22.67	Rutin	5.22	Standard
5	28.67	Pyrogallol	2.53	Standard
6	30.88	Mandalic acid	2.82	Standard
7	35.49	Phloroglucinol	1.53	Standard
8	36.31	Hydroxy benzoic acid	0.926	Standard
9	4.5	Gallic acid derivative	0.88	Fischer et al. [19]
10	13.9	Quercetin-3- glucoside	8.15	Santos et al. [20]

Where

C_X =_ Concentration of unknown

A_S_ = Peak area of standard

A_x_ = Peak area of unknown

Cs = Concentration of standard

The phenolic compounds identified in ethyl acetate fraction (Additional file 1: Figure S3) were given numbers **3**, **4**, **5**, **7** and **8**. Compound **3**, eluted at retention time 12.76 min, was morin with concentration 3.13 mg/100 g. Compound **4** was identified as rutin which was eluted at retention time of 22.67 min (concentration = 5.22 mg/100 g). Compound **5** was identified as pyrogallol and was eluted at a retention time of 28.67 min (concentration = 2.53 mg/100 g). Compounds **7**, and **8** eluted at retention times of 35.49 and 36.31 min were identified as phloroglucinol and hydroxy benzoic acid (concentrations 1.53 and 0.926 mg/100 g respectively). In n-butanol, n-hexane, chloroform, and aqueous fractions the targeted compound peaks were absent however, some peaks of unknown compound were there which were not possible to identify from the standard chromatogram through retention time and spectra. Gallic acid derivative and quercetin-3-glucoside were identified in them by comparison of their retention with already reported work in literature [20, 21]. The molecular structures of identified compounds are given in Additional file 1: Figure S4.

### Isolation of phenolic compounds from *Bergenia ciliata* rhizome

The silica gel column was eluted successively, using ethyl acetate and n-hexane solvent system with increasing polarities from 1 to 50%. Different fractions were combined according to their TLC profile and visualized by UV light. Twenty sub fractions designated as C-1 to C-20 were obtained which were re-subjected to silica gel column. The isolated and purified compounds were again subjected to HPLC analysis. A single broad peak confirms their purity and isolation. The three compounds were isolated and further characterized by FTIR and H-NMR. Their details are given below:

#### Compound 5: pyrogallol (Benzene-1, 2, 3-triol)

The compound **5**, pyrogallol (Benzene-1, 2, 3-triol) was isolated from the ethyl acetate extract as white, lustrous crystals. Its molecular formula is C_6_H_6_O_3_ (Additional file 1: Figure S5), UV/Visible maximum absorption peak is at 275 nm and melting point is 133 °C. The HPLC chromatogram was developed to confirm their purity and isolation (Additional file 1: Figure S6). Its infrared (FTIR) absorption bands indicated the presence of hydroxyl group at 3537 cm^− 1^, the peak at 3050 cm^− 1^ presents C-H, aromatic and C=C bond stretching at 1622 cm^− 1^ shows the presence of the aromatic nucleus (Additional file 1: Figure S7).

In ^1^H-NMR spectra, the triplets signal at 6.4 ppm were assigned to proton at C-1 and the duplets signal appeared at 6.2 ppm were due to aromatic proton at C-2 respectively. In the same way the duplets signal was there at 4.8 ppm for C-OH (Additional file 1: Figure S8). The present data was in close agreement with the previously reported data [23].

#### Compound 4: rutin (3, 3, 4, 5, 7-pentadroxyflavones-3-rutinoside)

Compound **4**, isolated as a light yellow powder from the ethyl acetate fraction was identified as rutin. Its molecular formula is C_27_ H_30_ O_16_ (Additional file 1: Figure S9), UV/Vis range = 304 nm and Melting Point = 193 °C. The HPLC analysis showed pure and broad peak of rutin confirmed its isolation and purity (Additional file 1: Figure S10). The structure of compound **4** was elucidated with the help of FTIR and ^1^H-NMR and comparing its spectral and physical data with the already reported data. The IR spectrum showed absorption peaks for hydroxyl group at 3417 cm^− 1^, the peak at 3050 cm^− 1^ confirmed the aromatic C-H group while peak at 1745 cm^− 1^ was for the carbonyl functionality. The peak at 2935 cm^− 1^ presenting the C-H while C=C double bond stretching at 1600 cm^− 1^ indicating an aromatic nucleus (Additional file 1: Figure S11).

In the H^1^-NMR Spectrum the multiplet signal appeared in the range 3.72–3.35 ppm were assigned to glucose protons and the duplets peak at 3.9, 1.6 ppm indicate the saturated proton in glucose molecule. In the same way the glucose proton duplets peak appears at 4.3 ppm of C-1. Similarly the duplet signals at 6.1, 6.3, 6.6 and 7.8 ppm indicated the aromatic protons at C-6, C-8, C-5 and C-2 respectively (Additional file 1: Figure S12). The present data was in close agreement to the previously reported data [24].

#### Compound 3: morin (2-(2, 4-Dihydroxyphenyl)-3, 5, 7-trihydroxychromen-4-one)

Compound **3**; morin was isolated from the ethyl acetate extract as yellow powder. Its molecular formula is C_15_H_10_O_7_ (Additional file 1: Figure S13), UV/Vis range = 263 nm and melting point = 299–300 °C. HPLC chromatogram of the isolated compound morin was also obtained which confirm its purity and isolation (Additional file 1: Figure S14). The FTIR absorption bands at 3430 cm^− 1^ confirmed the presence of hydroxyl group and C=C double bond stretching at 1653 cm^− 1^ representing the aromatic nuclei. The peak at 2981 cm^− 1^ shows the aromatic C-H and the peak at 1739 cm^− 1^ represent carbonyl functionality (Additional file 1: Figure S15).

The duplets signal at 6.21 and 6.46 ppm in ^1^H-NMR spectra indicates the aromatic proton at C-6 and C-8 respectively. While the signals at 7.53, 6.70, and 7.40 ppm indicating the aromatic proton of ring B at C-3, C-5 and C-6 respectively. In the same way the duplets signal appeared at 4.78 ppm was assigned to C-OH at C-5, C-7 in the ring A as well as for C-2 and C-4 in ring B respectively. The singlet signal at 12.86 ppm presenting the C-OH proton at C-3 (Additional file 1: Figure S16). The present data was in close agreement with the reported research in literature [25].

### Antioxidant potential of isolated compounds

The phenolic compounds; rutin, morin and pyrogallol isolated from the rhizome of *Bergenia ciliata* showed a high antioxidant potentials against DPPH and ABTS free radical when tested at different concentration ranging from 1000 to 62.5 μg/ml. Against DPPH, the percent free radical scavenging potential were found higher for pyrogallol with IC_50_ value of 68 μg/ml followed by morin with IC_50_ 105 μg/ml. Rutin also showed moderate activity with IC_50_ of 150 μg/ml. Against the ABTS free radical, with IC_50_ = 90 μg/ml, pyrogallol was more potent followed by morin (IC_50_ = 145 μg/ml). Ascorbic acid was used as a standard and its IC_50_ value was 50, 62 μg/ml against DPPH and ABTS free radicals respectively (Table 5 and Fig. 2). According to literature, the number and position of hydroxyl (−OH) and methoxy (−OCH_3_) groups in the phenolic acid plays an important role in the scavenging of free radicals [26].

**Table 5:** Percent ABTS and DPPH radical scavenging potential of pyrogallol, morin and rutin isolated from *Bergenia ciliata* rhizome using ascorbic acid as standard

Samples	Concentrations (μg/mL)	DPPH percent inhibition (mean ± S.E.M)	DPPH IC_50_ (μg/mL)	ABTS percent inhibition (mean ± S.E.M)	ABTS IC_50_ (μg/mL)
+Pyrogallol	1000	89.35 ± 0.92 ^ns^		87.02 ± 0.81 ^ns^	
	500	81.64 ± 1.33 ^ns^		77.81 ± 1.09 ^ns^	
	250	70.30 ± 1.05 ^ns^	68	66.42 ± 0.90 ^ns^	85
	125	59.93 ± 2.18 ^ns^		53.26 ± 1.62**	
	62.5	48.72 ± 0.89 ^ns^		39.60 ± 1.39***	
Morin	1000	83.93 ± 0.72***		80.86 ± 1.09***	
	500	72.72 ± 1.56**		70.67 ± 1.02***	
	250	60.25 ± 2.28 ***	105	58.04 ± 2.44***	145
	125	48.72 ± 1.11 ^ns^		44.15 ± 0.56***	
	62.5	40.85 ± 0.94*		30.50 ± 1.43***	
Rutin	1000	80.90 ± 1.27***		78.33 ± 0.63***	
	500	68.43 ± 1.40***		69.05 ± 0.64***	
	250	57.47 ± 0.93***	150	57.56 ± 1.42***	183
	125	46.63 ± 1.17***		44.30 ± 0.79***	
	62.5	35.38 ± 2.28***		29.75 ± 2.18***	
Ascorbic acid	1000	92.67 ± 0.73		89.94 ± 0.86	
	500	80.45 ± 0.96		80.29 ± 0.79	
	250	71.34 ± 2.16	50	68.93 ± 0.45	62
	125	62.90 ± 0.85		58.90 ± 0.48	
	62.5	53.78 ± 0.76		50.88 ± 0.32	

Ascorbic acid was used as a positive control. Data is represented as (mean ± S.E.M) n = 3. Values significantly different as compared to positive control, *: *P* < 0.05, **: *P* < 0.01, ***: *P* < 0.001, ns: *P* > 0.05

### AChE and BChE inhibition potential of isolated compounds

The isolated compounds rutin, morin and pyrogallol inhibited AChE and BChE activities when treated with compound solutions in range of 1000 to 62.5 μg/ml. Against AChE, the percent inhibition potential was higher for pyrogallol with IC_50_ value of 28 μg/ml followed by morin with IC_50_ 64 μg/ml while for rutin the IC_50_ was 118 μg/ml. Against the BChE the IC_50_ of the isolated compounds; pyrogallol, morin and rutin were 70, 116 and 172 μg/ml respectively (Table 6). Galanthamine was used as a standard and its IC_50_ value was 24, 54 μg/ml against AChE and BChE respectively.

**Table 6:** Percent AChE and BChE inhibition potential of pyrogallol, morin and rutin isolated from *Bergenia ciliata* rhizome

Samples	Concentrations (μg/mL)	AChE percent inhibition (mean ± S.E.M)	AChE IC_50_ (μg/mL)	BChE percent inhibition (mean ± S.E.M)	BChE IC_50_ (μg/mL)
Pyrogallol	1000	94.35 ± 0.72 ^ns^		92.02 ± 1.89 ^ns^	
	500	87.40 ± 1.51 ^ns^		80.35 ± 0.30 *	
	250	80.12 ± 0.62 ^ns^	28	71.18 ± 1.38 ^ns^	70
	125	66.24 ± 2.14 ^ns^		58.28 ± 0.93 ^ns^	
Morin	1000	85.51 ± 3.19***		80.46 ± 0.76***	
	500	77.23 ± 0.91***		68.44 ± 2.22***	
	250	68.82 ± 0.80 ***	64	60.51 ± 0.50***	116
	125	56.33 ± 1.45 ***		51.13 ± 0.56***	
Rutin	1000	75.11 ± 1.41***		72.05 ± 0.86***	
	500	66.72 ± 0.62***		62.47 ± 1.72***	
	250	60.20 ± 2.35***	118	56.68 ± 0.70***	172
	125	48.09 ± 0.64***		45.00 ± 3.79***	
Galanthamine	1000	97.24 ± 0.74		92.26 ± 0.92	
	500	90.43 ± 0.50		86.51 ± 0.77	
	250	79.00 ± 0.65	24	71.80 ± 0.48	54
	125	66.07 ± 1.46		62.43 ± 0.39	

Galanthamine was used as a positive control. Data is represented as (mean ± S.E.M) n = 3. Values significantly different as compared to positive control, *: *P* < 0.05, **: *P* < 0.01, ***: *P* < 0.001, ns: *P* > 0.05

## Conclusion

In the present study three bioactive compounds; pyrogallol, morin and rutin were successfully isolated in pure form for the first time from *Bergenia ciliata*. The identification and quantification of these compounds was carried out using reverse phase HPLC, NMR and FTIR techniques. The isolated compounds showed potent antioxidant activities against DPPH and ABTS free radicals. The crude extract and sub-fractions were also tested for their antioxidant and enzyme inhibitory potentials. The total phenolic and flavonoid contents were also determined. From the results it was concluded that this plant could be used in the form of its extract or as such for the treatment of oxidative stress and neurological disorders. Further studies are needed to evaluate the plant for other medicinal properties as well.

## Supplementary information

**Additional file 1: Figure S1**. Representative HPLC-UV chromatograms of the standard compounds at 320 nm. **Figure S2**. HPLC chromatogram of crude extract of *B. ciliata* rhizome. **Figure S3.** Chromatogram of ethyl acetate fraction of *B. ciliata* rhizome. **Figure S4**. Structures of phenolic compounds identified through using HPLC-UV analysis in crude extract and ethyl acetate fraction of *Bergenia ciliata* rhizome. **Figure S5**. Chemical structure of pyrogallol isolated from *Bergenia ciliata* rhizome. **Figure S6.** HPLC Chromatogram of the isolated pyrogallol. **Figure S7.** FTIR spectra of the isolated compound pyrogallol. **Figure S8.** H^1^-NMR spectra of the isolated compound pyrogallol. **Figure S9.** Chemical structure of rutin isolated from *Bergenia ciliata* rhizome. **Figure S10.** HPLC Chromatogram of the isolated rutin. **Figure S11.** FTIR Spectra of the isolated compound rutin. **Figure S12.** H^1^-NMR Spectra of the isolated compound rutin. **Figure S13.** Structure of morin isolated from *Bergenia ciliata* rhizome. **Figure S14**. HPLC Chromatogram of the isolated morin. **Figure S15.** FTIR Spectra of the isolated compound morin. **Figure S16**. H^1^-NMR Spectra of the isolated compound morin

## Data Availability

The datasets used and/or analysed during the current study available from the corresponding author on reasonable request.

## Abbreviations

ABTS: 2,2′-Azino-Bis-3 Ethylbenzothiazoline-6-Sulfonic Acid
AChE: Acetylcholine esterase
*B. ciliata*: *Bergenia ciliata*
BChE: Butyryl choline esterase
DPPH: 2,2-Diphenyle-1 picrylhydrazyle
DTNB: 5,5-dithio-bis-nitrobenzoic acid
HPLC: High performance liquid chromatography
