# Supplementary material for: Isolation of bioactive compounds from Bergenia ciliata (haw.) Sternb rhizome and their antioxidant and anticholinesterase activities
Source: BMC Complement Altern Med. 2019 Nov 6;19:296. doi: 10.1186/s12906-019-2679-1 (PMC6833214; doi:10.1186/s12906-019-2679-1)
Supplement: Supplementary file 1 — Additional file 1: Figure S1. Representative HPLC-UV chromatograms of the standard compounds at 320 nm. Figure S2. HPLC chromatogram of crude extract of B. ciliata rhizome. Figure S3. Chromatogram of ethyl acetate fraction of B. ciliata rhizome. Figure S4. Structures of phenolic compounds identified through using HPLC-UV analysis in crude extract and ethyl acetate fraction of Bergenia ciliata rhizome. Figure S5. Chemical structure of pyrogallol isolated from Bergenia ciliata rhizome. Figure S6. HPLC Chromatogram of the isolated pyrogallol. Figure S7. FTIR spectra of the isolated compound pyrogallol. Figure S8. H1-NMR spectra of the isolated compound pyrogallol. Figure S9. Chemical structure of rutin isolated from Bergenia ciliata rhizome. Figure S10. HPLC Chromatogram of the isolated rutin. Figure S11. FTIR Spectra of the isolated compound rutin. Figure S12. H1-NMR Spectra of the isolated compound rutin. Figure S13. Structure of morin isolated from Bergenia ciliata rhizome. Figure S14. HPLC Chromatogram of the isolated morin. Figure S15. FTIR Spectra of the isolated compound morin. Figure S16. H1-NMR Spectra of the isolated compound morin [file 12906_2019_2679_MOESM1_ESM.docx]

**Additional files**


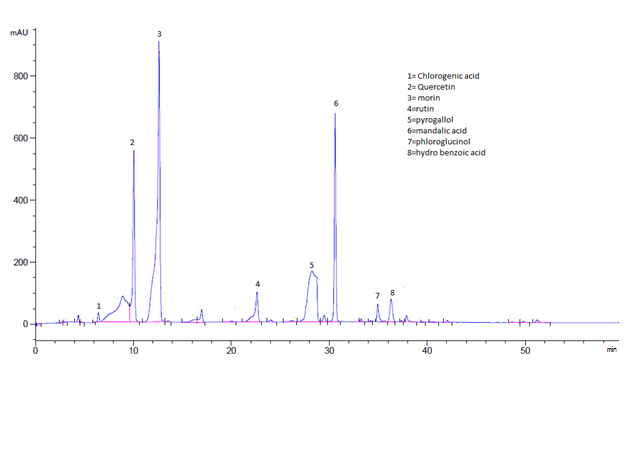


**Figure S1**: Representative HPLC-UV chromatograms of the standard compounds at 320 nm.


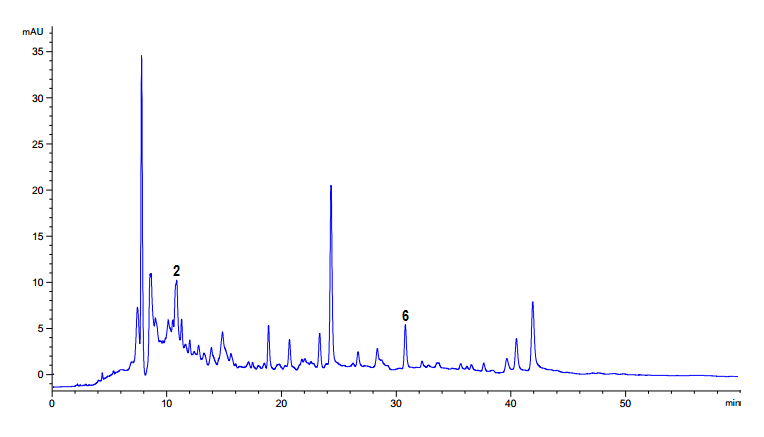


**Figure S2**: HPLC chromatogram of crude extract of *B. ciliata* rhizome.


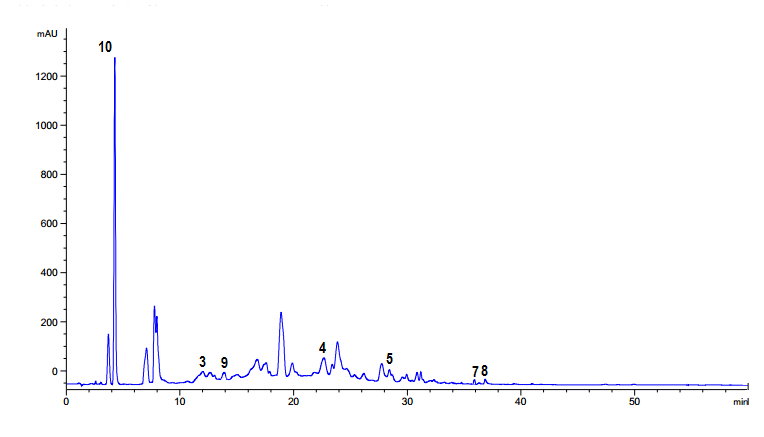


**Figure S3:** Chromatogram of ethyl acetate fraction of *B. ciliata* rhizome

| 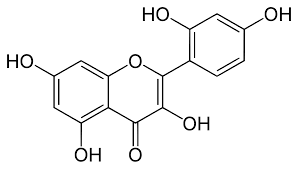  Morin | 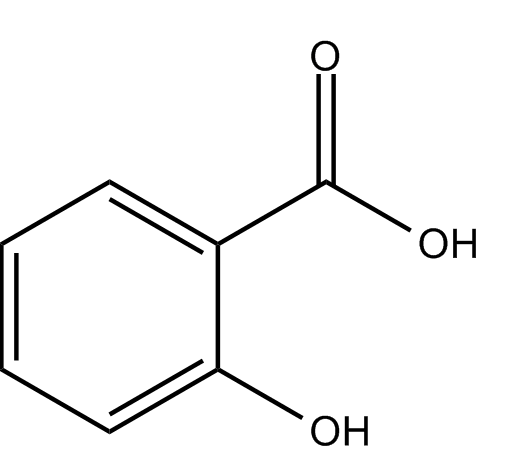  Hydroxy benzoic acid |
| --- | --- |
| 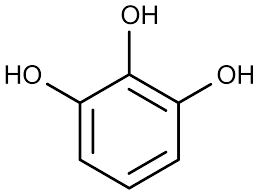  Pyrogallol | 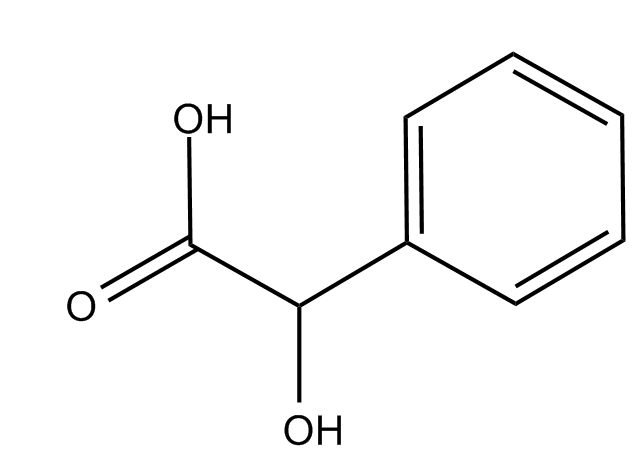  Mandalic acid |
| 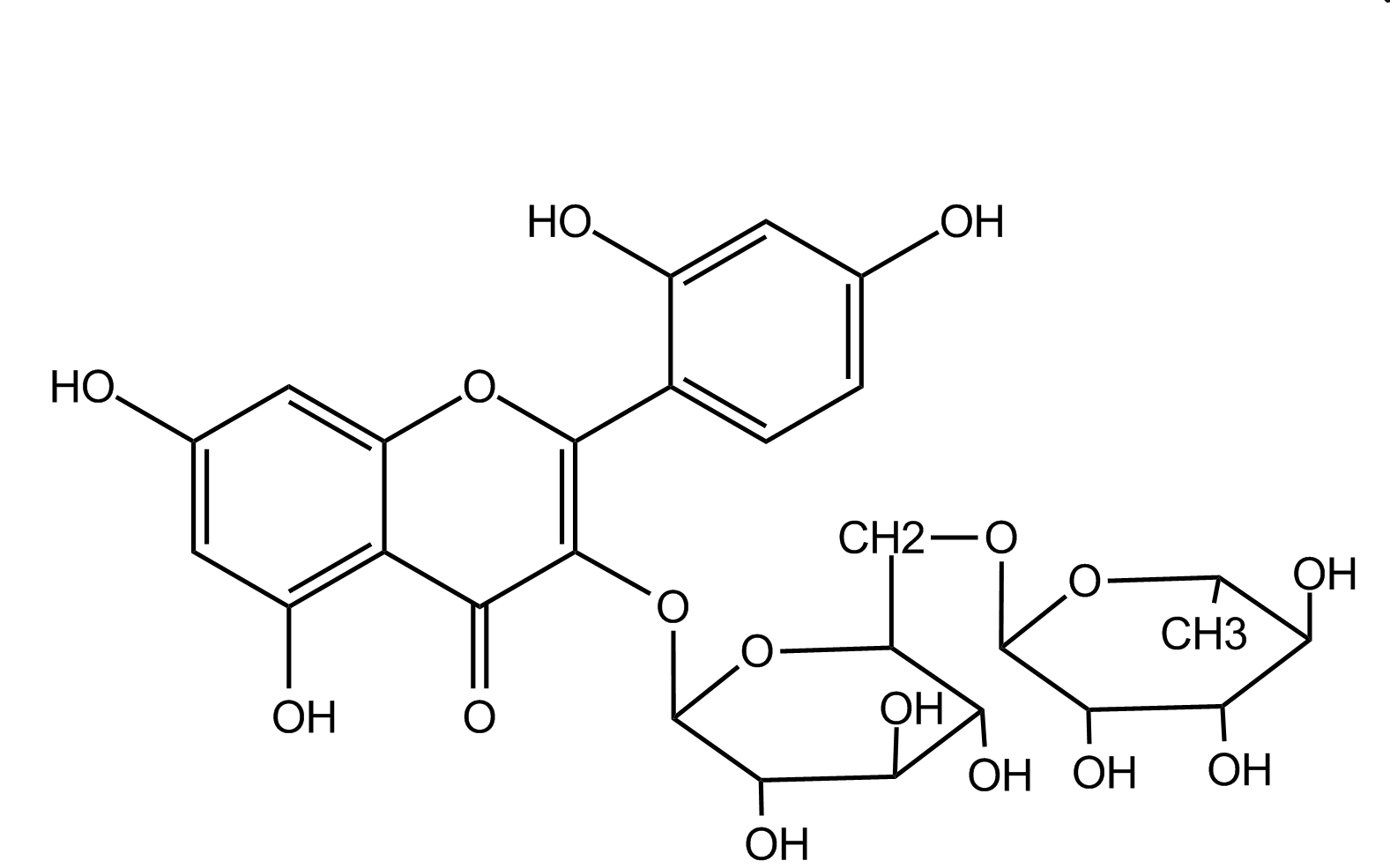  Rutin | 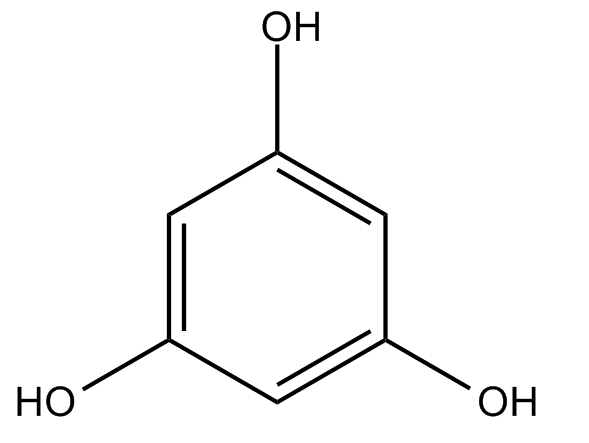  Phloroglucinol |
| 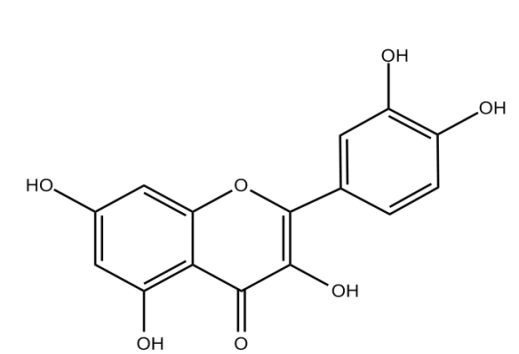Quercetin | 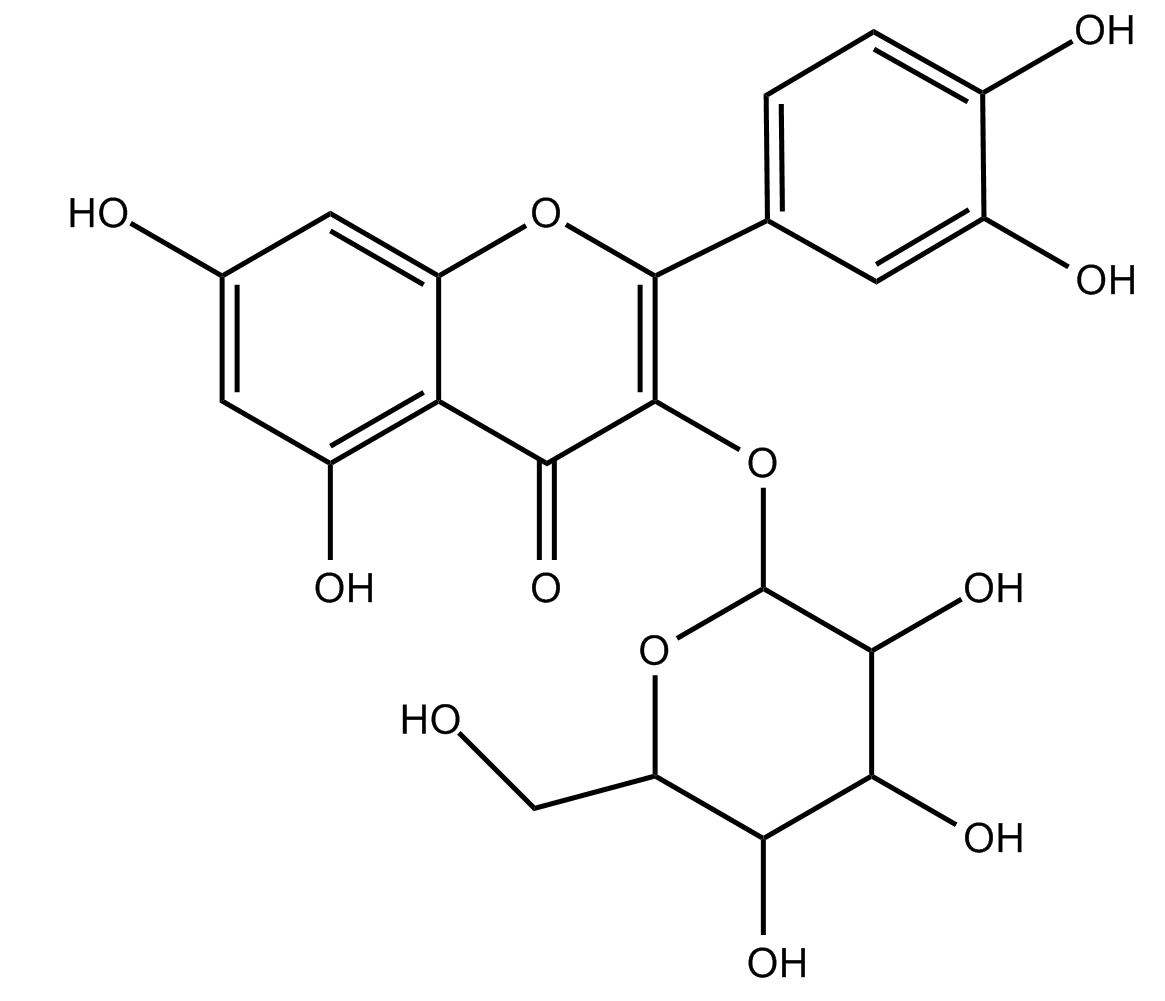  Quercetin-3-glucoside |

**Figure S4**: Structures of phenolic compounds identified through using HPLC-UV analysis in crude extract and ethyl acetate fraction of *Bergenia ciliata* rhizome.


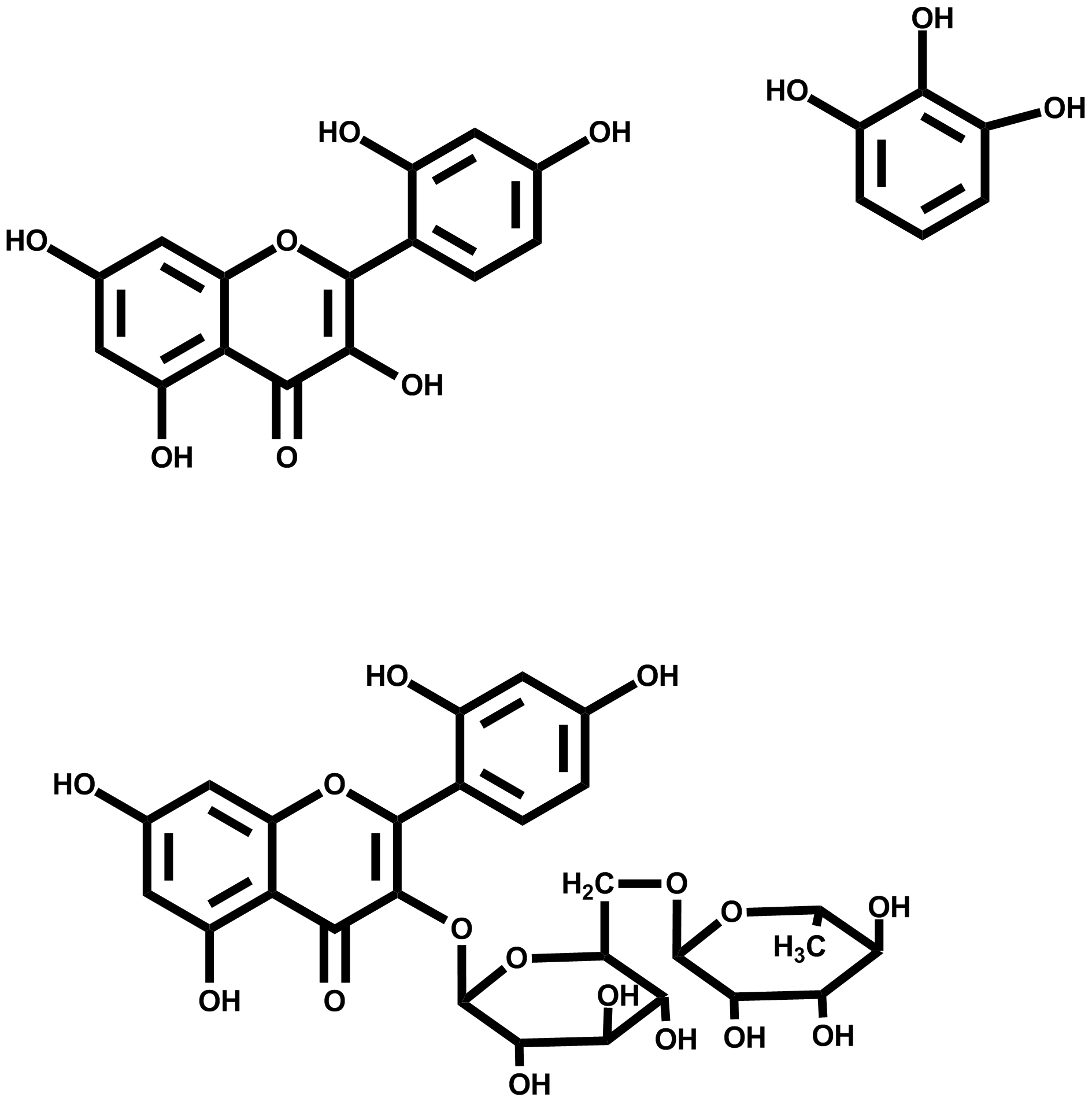


**Figure S5**: Chemical structure of pyrogallol isolated from *Bergenia ciliata* rhizome


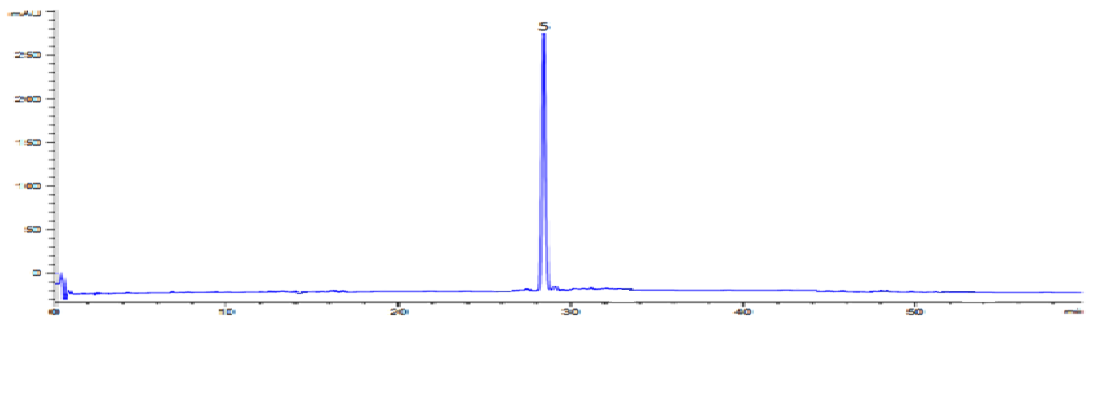
**Figure S6:** HPLC Chromatogram of the isolated pyrogallol

**
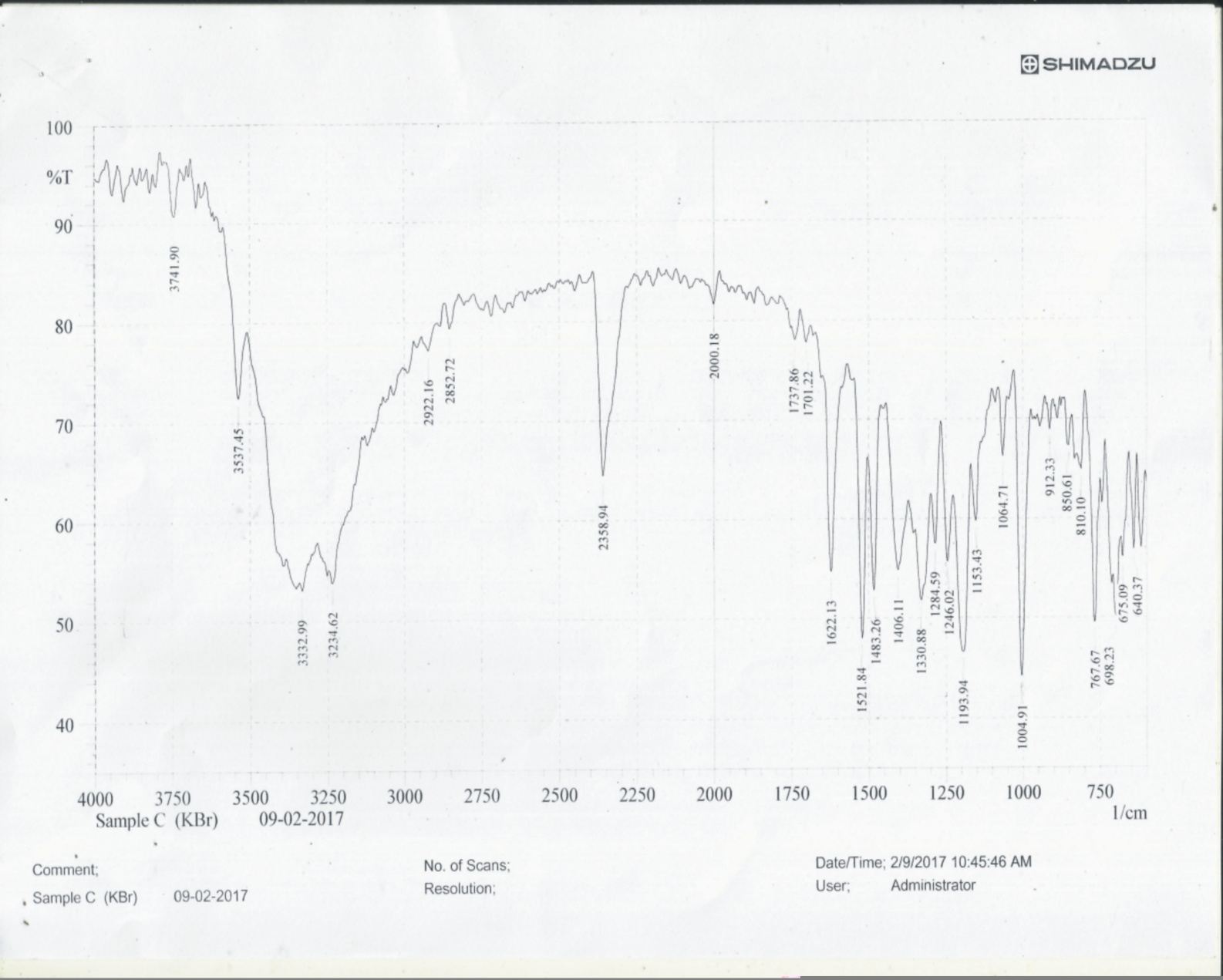
Figure S7:** FTIR spectra of the isolated compound pyrogallol

**
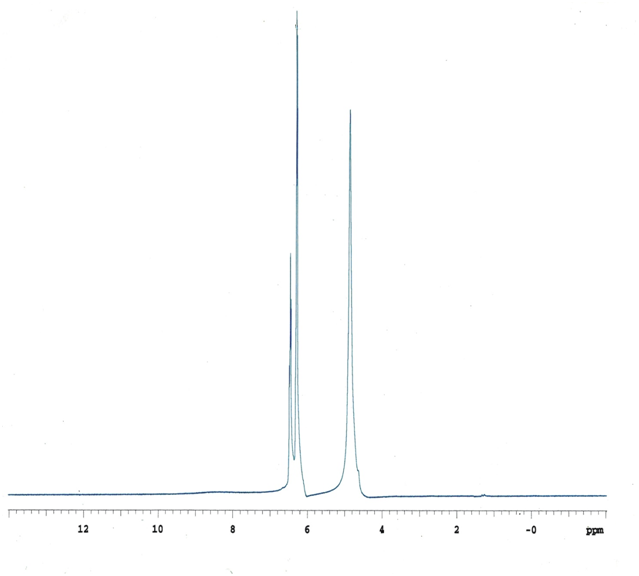
Figure S8:** H^1^-NMR spectra of the isolated compound pyrogallol


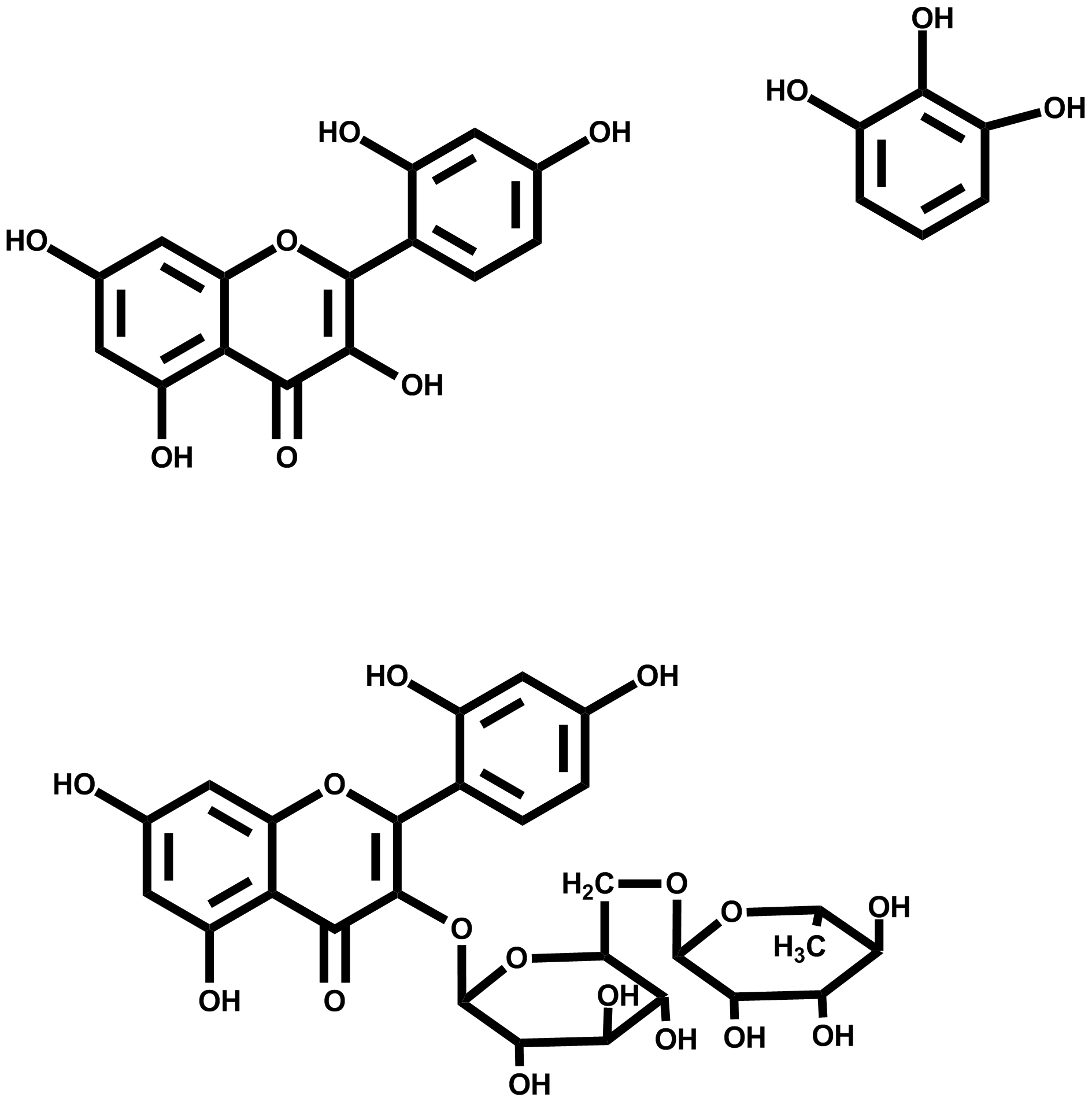


**Figure S9**: Chemical structure of rutin isolated from *Bergenia ciliata* rhizome.


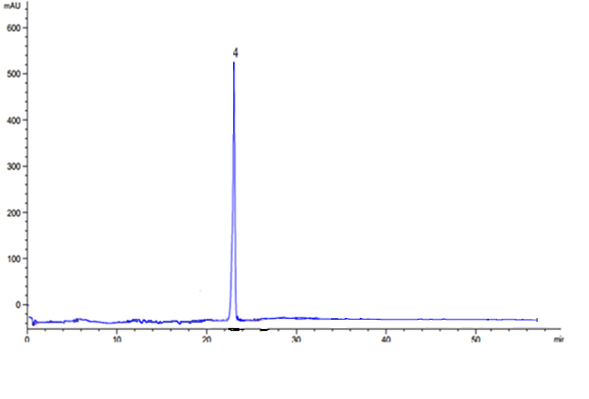
**Figure S10:** HPLC Chromatogram of the isolated rutin.


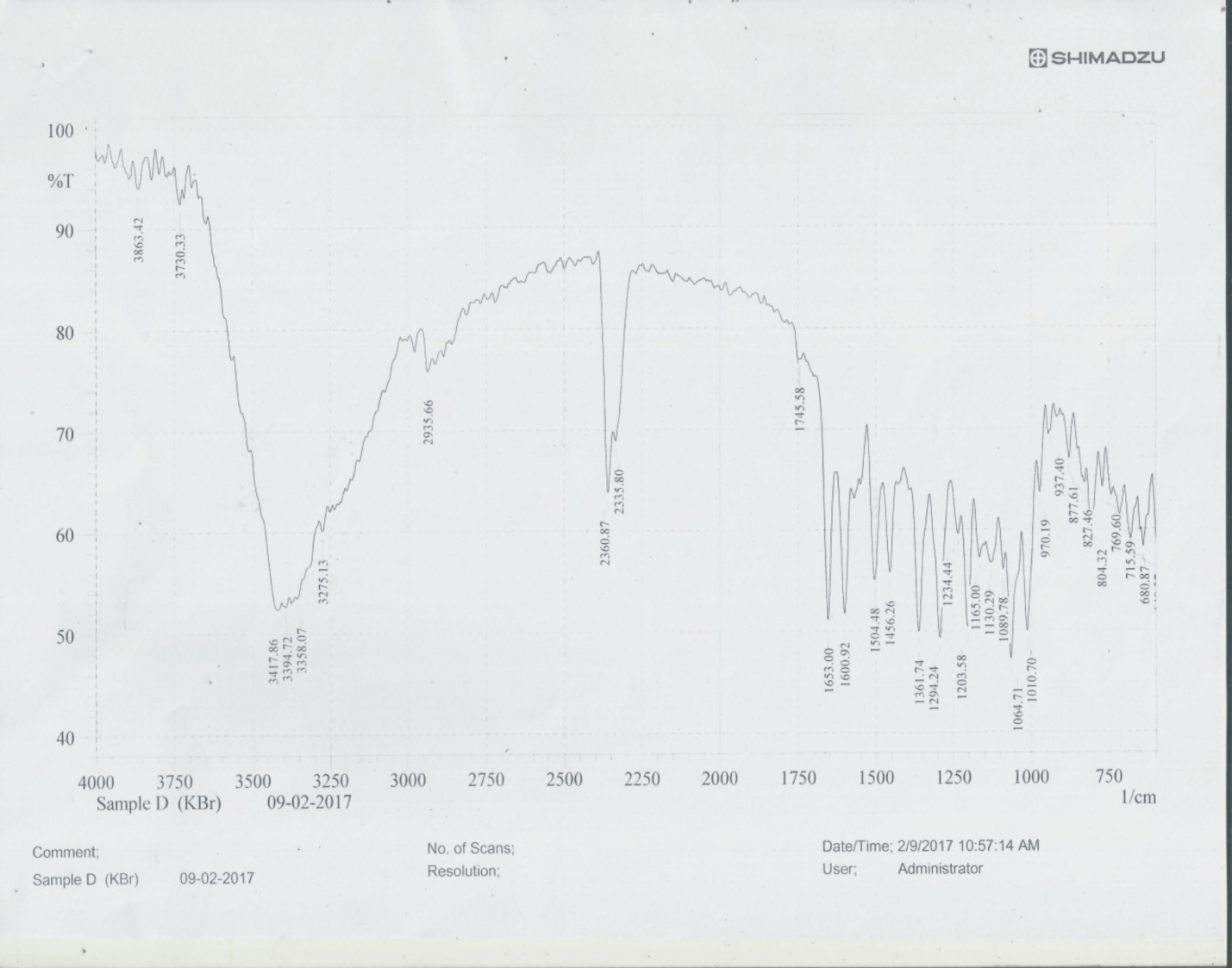
**Figure S11:** FTIR Spectra of the isolated compound rutin


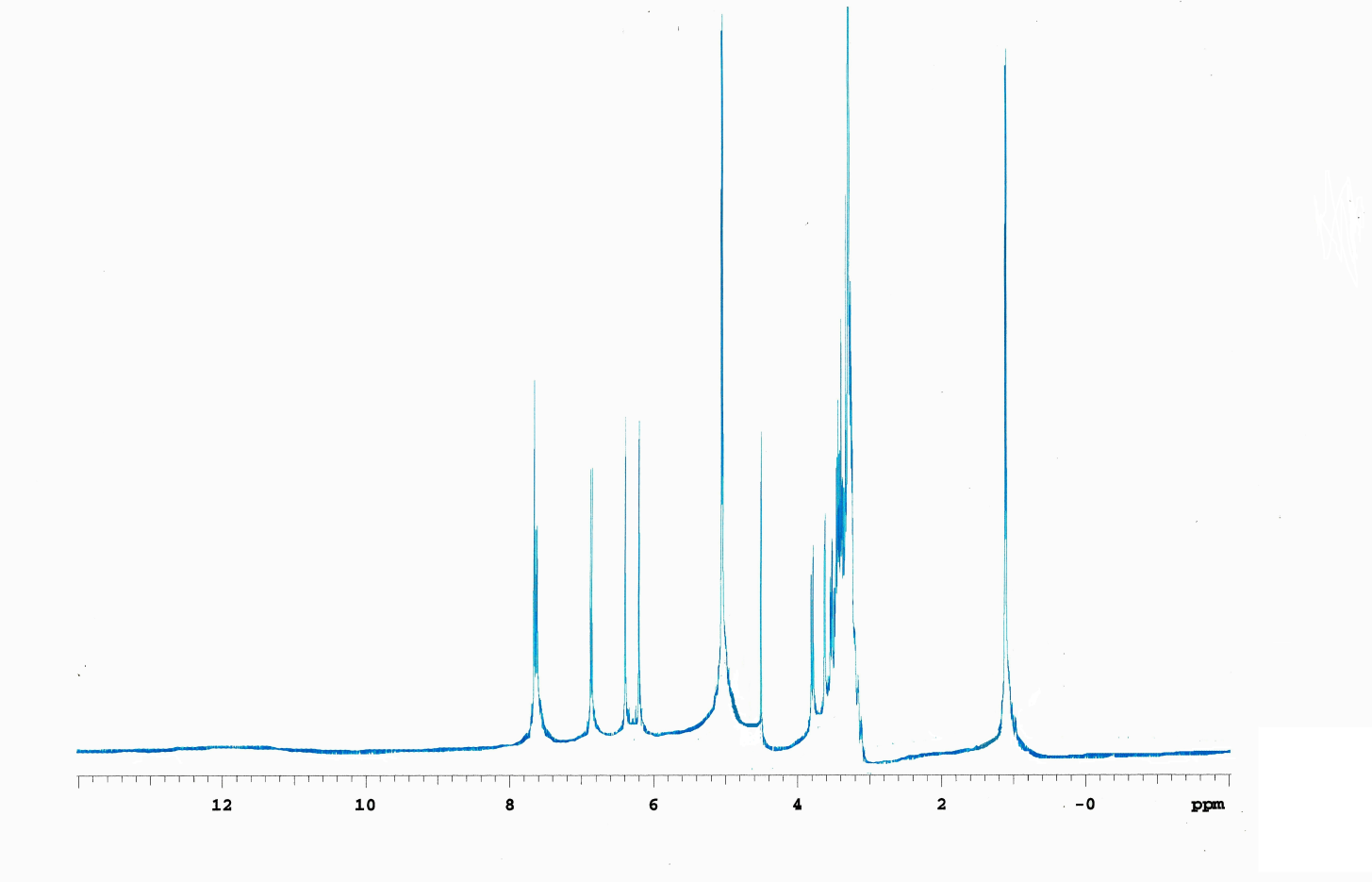
**Figure S12**: H^1^-NMR Spectra of the isolated compound rutin


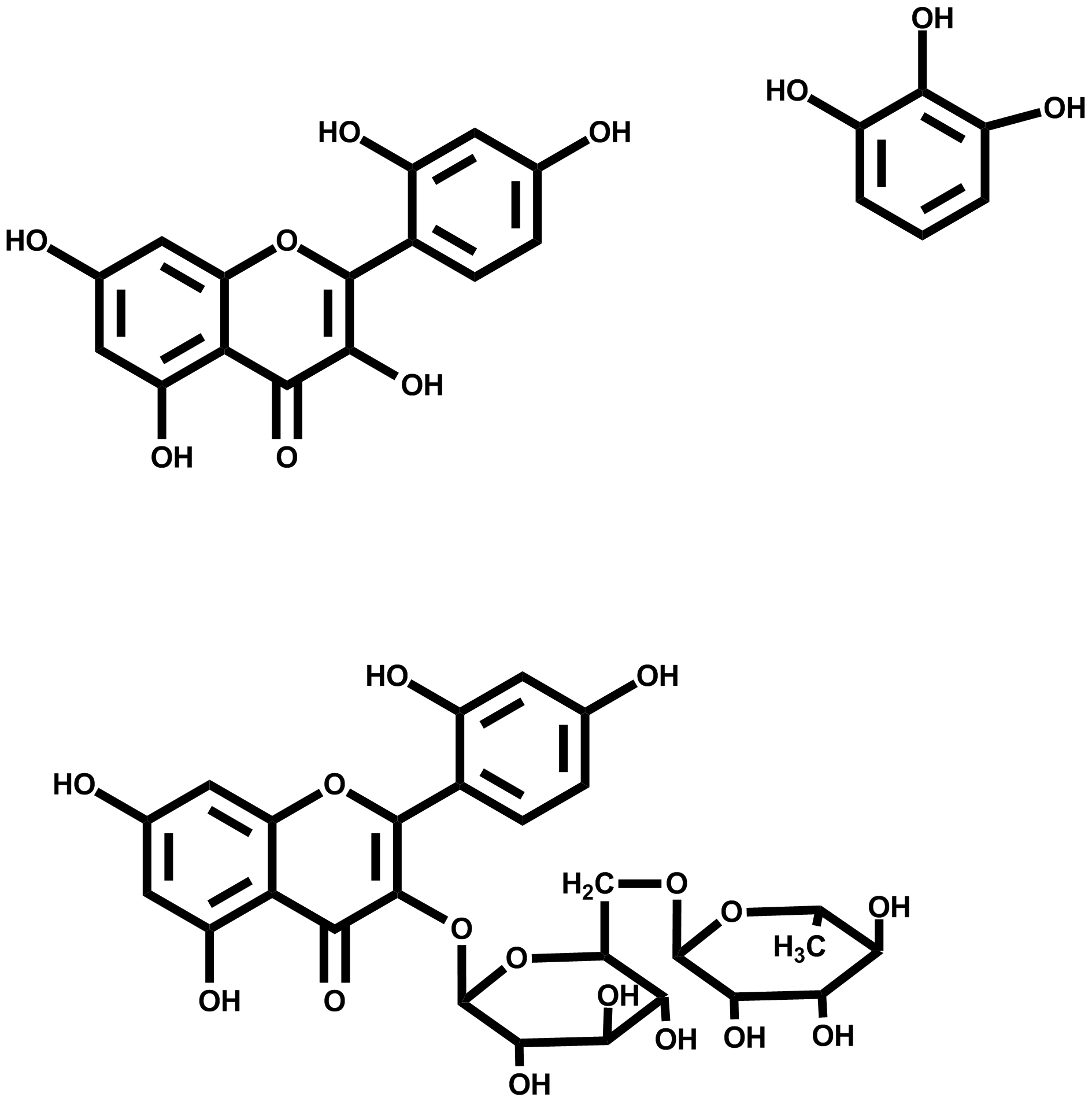


**Figure S13**: Structure of morin isolated from *Bergenia ciliata* rhizome.


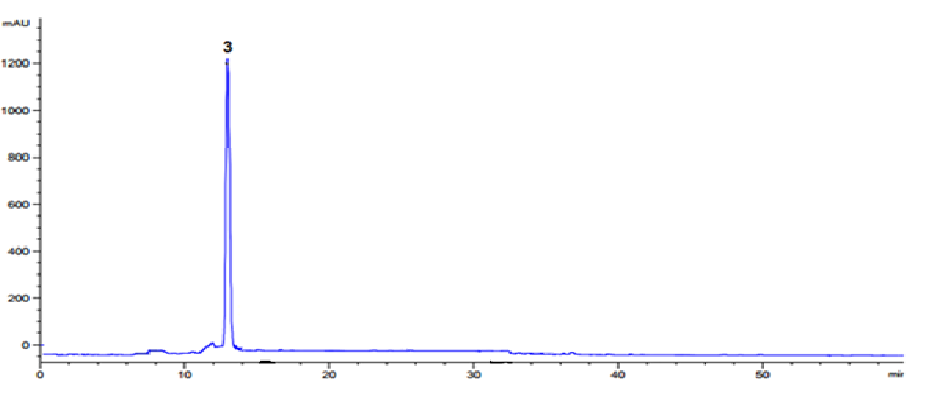
**Figure S14**: HPLC Chromatogram of the isolated morin.

**
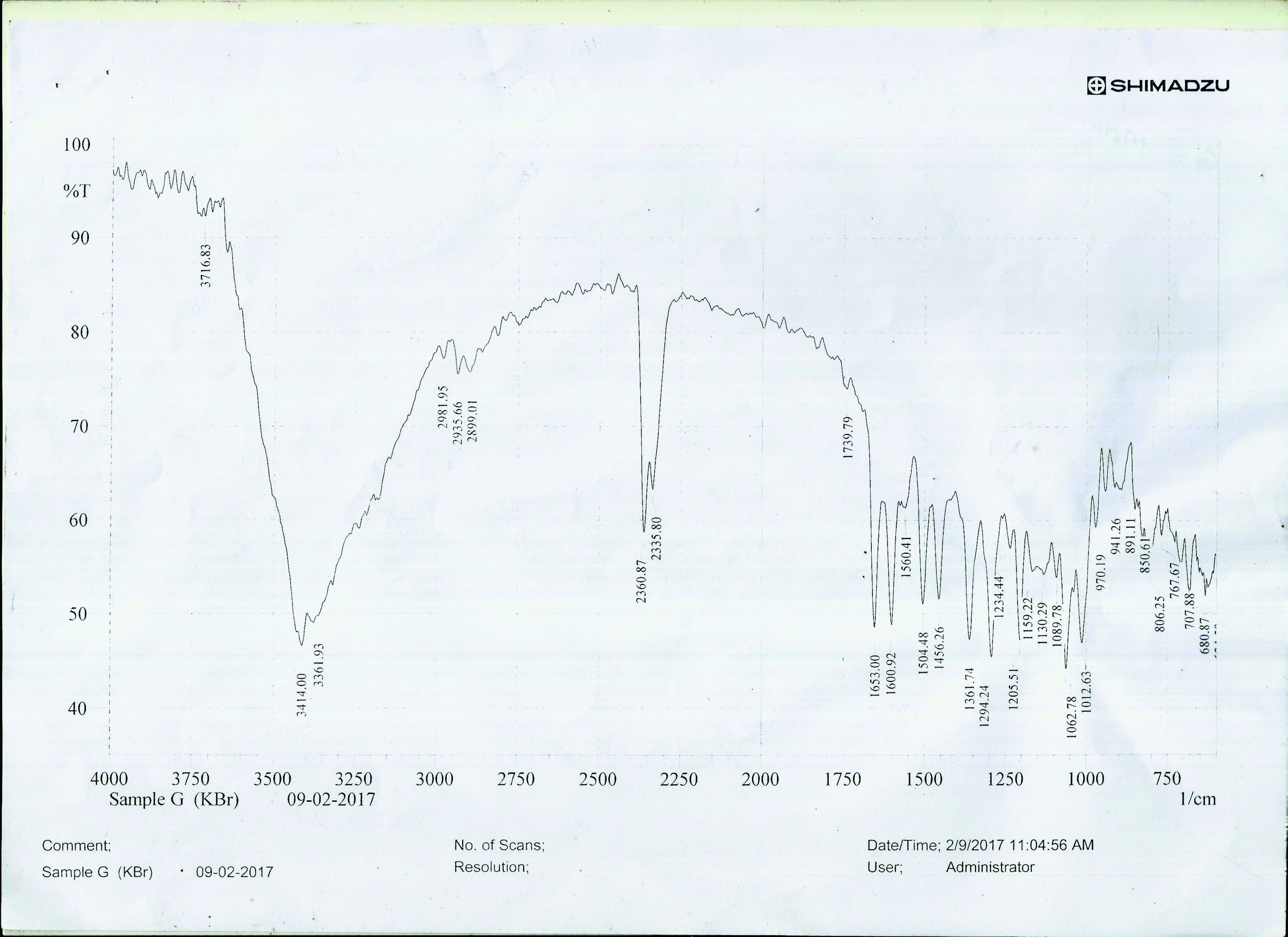
Figure S15**: FTIR Spectra of the isolated compound morin

**
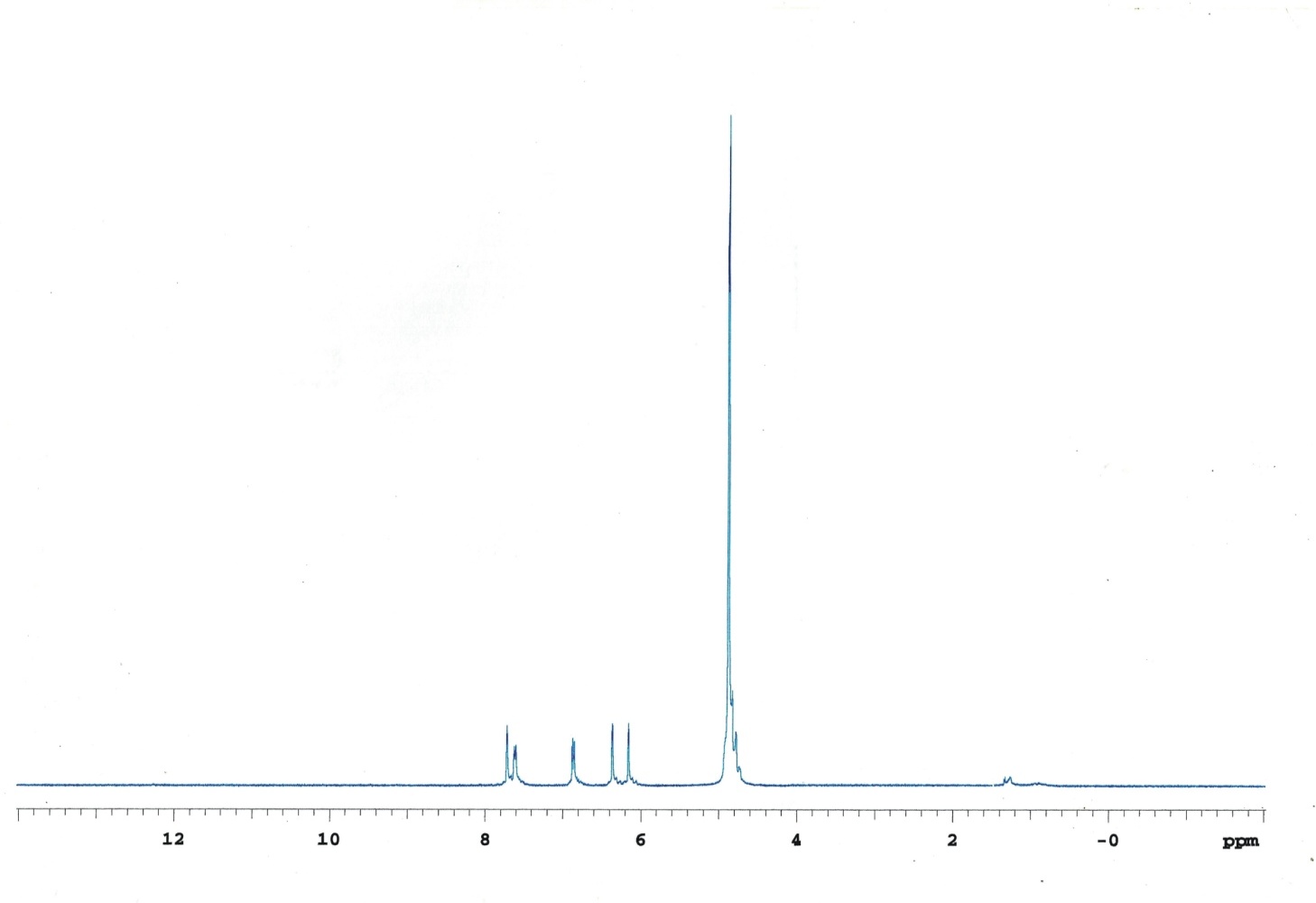
Figure S16**: H^1^-NMR Spectra of the isolated compound morin
